# Local Expansion of a Panmictic Lineage of Water Bloom-Forming Cyanobacterium *Microcystis aeruginosa*

**DOI:** 10.1371/journal.pone.0017085

**Published:** 2011-02-24

**Authors:** Yuuhiko Tanabe, Makoto M. Watanabe

**Affiliations:** Graduate School of Life & Environmental Sciences, University of Tsukuba, Tsukuba, Ibaraki, Japan; J. Craig Venter Institute, United States of America

## Abstract

In previous studies, we have demonstrated that the population structure of the bloom-forming cyanobacterium *Microcystis aeruginosa* is clonal. Expanded multilocus sequence typing analysis of *M. aeruginosa* using 412 isolates identified five intraspecific lineages suggested to be panmictic while maintaining overall clonal structure probably due to a reduced recombination rate between lineages. Interestingly, since 2005 most strains belonging to one of these panmictic clusters (group G) have been found in a particular locality (Lake Kasumigaura Basin) in Japan. In this locality, multiple, similar but distinct genotypes of this lineage predominated in the bloom, a pattern that is unprecedented for *M. aeruginosa*. The population structure underlying blooms associated with this lineage is comparable to epidemics of pathogens. Our results may reveal an expansion of the possible adaptive lineage in a localized aquatic environment, providing us with a unique opportunity to investigate its ecological and biogeographical consequences.

## Introduction

The water bloom-forming cyanobacterium *Microcystis aeruginosa* is a unicellular, colony-forming cyanobacterium distributed worldwide in eutrophic freshwater environments (lakes, ponds, and reservoirs) [1]. The occurrence of *Microcystis* water blooms has resulted in severe environmental problems including the release of bad odors and bottom layer anoxia. However, the most serious problem associated with *Microcystis* blooms is the production of hepatotoxic cyanotoxins called microcystins. Accidental exposure to microcystin-contaminated water causes acute poisoning in humans and livestock [2], [3].

To genetically characterize *Microcystis* isolates in detail, we developed a multilocus sequence typing (MLST) scheme using seven housekeeping loci [4]. Our previous MLST analyses indicated that *M. aeruginosa* is divided into at least seven distinct phylogenetic clusters [5] with partial correspondence to either colony morphology or microcystin production [6]. On the other hand, the discovery of an intraspecific lineage “group G” is notable because virtually all isolates belonging to this lineage were obtained from a location in Lake Kasumigaura, Japan, in July 2005 [5]. This finding motivates us to collect and genetically characterize many more isolates from this locality and nearby areas to assess whether this group is endemic.

It is widely recognized that recombination highly influences bacterial population structure [7]. The impact of recombination on genetic diversity of bacterial populations was first defined in the milestone work of Smith et al. [8], who proposed the “clonal,” “panmictic,” and “epidemic” population structures on the basis of linkage disequilibrium (LD) between multiple marker loci. Since then numerous microbial species have been characterized with regard to the degree of clonality on the basis of multilocus LD as well as by improved methodologies, revealing differing impacts of recombination on the genetic diversity among species [9], [10]. Recombination has also been suggested to be an important factor in diversification of cyanobacterial species [11]–[13], and several studies have addressed the degree of clonality within them. Studies indicated that the Baltic Sea and North Sea populations of the marine cyanobacteria *Microcoleus chthonoplastes* [14] are panmictic, whereas the Baltic Sea population of *Nodularia* [15] and the Japanese population of *M. aeruginosa* [4] are clonal or weakly clonal. However, a clonal population structure does not indicate the absence of recombination. A substantial level of recombination was observed for *M. aeruginosa* [4]. Furthermore, because all the above studies were based on the erroneous assumption that recombination occurs equally between within-species individuals regardless of the magnitude of genetic relatedness, it is possible that these analyses underestimated the frequency of recombination by regarding multiple freely recombining units as a single unit. The best way to overcome this inherent problem is by assessing recombination for each distinct intraspecific lineage. However, to date, few studies have assessed the lineage-specific recombination within cyanobacterial species [6].

Accumulation of MLST data allows us to examine group G and other within-species clusters of *M. aeruginosa* with regard to population genetics, particularly in relation to the impact of lineage-specific recombination and possible endemism. For these reasons, we expanded MLST data sets of *M. aeruginosa* to include 412 isolates representing 237 unique multilocus sequence types (STs).

## Results and Discussion

**Figure 1:**
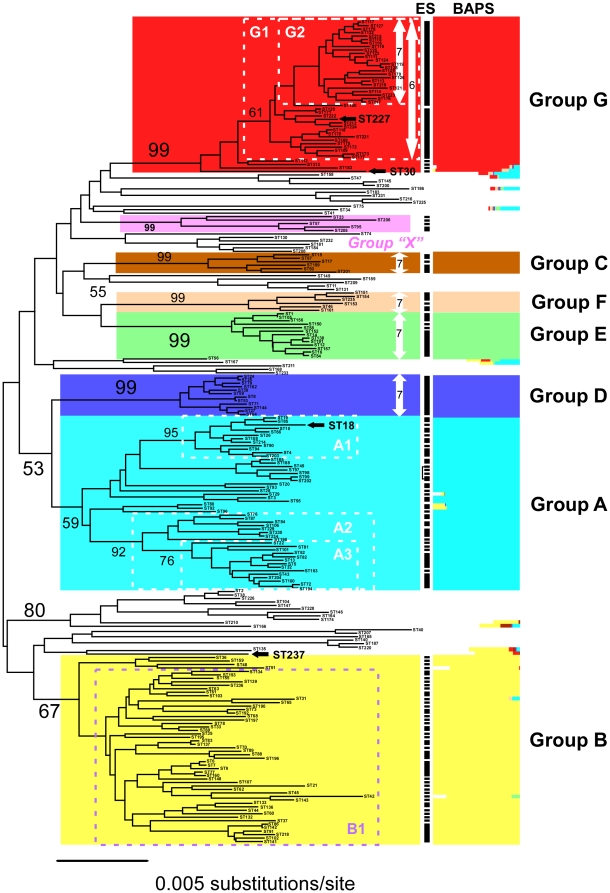
Phylogenetic tree of 237 MLST genotypes (STs) of *Microcystis aeruginosa*. Bootstrap statistical support was indicated in the major branches. Seven major groups (groups A–G [5]) and a putative hybrid group (group X, suggested by microcystin gene analyses [6]) were color-coded. Note that the hybrid nature of group X was not supported by the result of BAPS analysis of MLST genes. Arrows indicate the panmictic lineage with the number of loci used for LD analysis. The dotted boxes indicate the subset of that group analyzed for LD. The result of admixture analysis is indicated on the right side of the phylogenetic tree where each bar indicates the origin of gene segments of the color corresponding to the phylogenetic group defined by the MLST phylogeny. In addition to the three STs mentioned in the text, ST18 is highlighted in boldface because it represents NIES-843, for which the entire genome sequence is available [45]. The putative ecotypes identified by Ecotype Simulation analysis are also indicated within the black bar. Note that ST49, 99, and 202, which are paraphyletic in the NJ tree, were identified as members of the same ecotype, thus being connected by the black line.

We obtained 82 novel STs in addition to the 155 previously characterized ones [4]–[6] (see Table S1 for a more detailed description of novel isolates). Phylogenetic analysis of a collection of 237 STs of *M. aeruginosa* identified seven distinct lineages (groups A–G) with moderate to high statistical support (Fig. 1), which is consistent with our previous analysis [5]. On the other hand, Bayesian assignment analysis (BAPS [16]) identified eight groups; the same seven groups as identified by phylogenetic analysis and an additional group that encompassed all STs belonging to none of the above seven groups (indicated without color coding, Fig. 1). Because possible admixture events using BAPS analysis are more evident in this group than those in other groups (Fig. 1), the ambiguous phylogenetic assemblage of this group is possibly due to recombination between lineages. On the other hand, the result indicated that admixture across lineages appears to be relatively uncommon. This suggests the presence of a barrier to genetic exchange between lineages, although the boundaries are not completely established, as is the usual case for bacteria [17].

The differing impact of genetic exchange between and within each lineage was further highlighted by a range of statistical tests for recombination. Previous multilocus LD analysis with a limited number of strains (76 STs) suggested that the population structure of *M. aeruginosa* is clonal [4]. Overall clonality was again confirmed by analysis with 237 STs in which the standardized index of association (*I_A_^S^* [18]), which ranges from 0 (panmixia) to 1 (absolute LD), indicated a significant positive value (*P*<0.001, Table 1). However, LD analysis of strain subsets revealed the differing impact of recombination within *M. aeruginosa*. Five intraspecific lineages, groups C–F and a subgroup within group G (designated group G2), were suggested to be panmictic. Exclusion of *glnA*, which is highly polymorphic in group G (possessing a larger number of alleles relative to other loci; Table S1), extends the range of the freely recombining unit within group G to include more STs (designated group G1) on the basis of *I_A_^S^*. Furthermore, results of maximum likelihood tests for tree congruence [9] and estimated parameter values for relative impact (*r*/*m*) and rate (*ρ*/*θ*) of recombination and mutation supported the recombinogenic nature of the five lineages (Table S2). The average nucleotide diversity (*π*) for the five lineages of *M. aeruginosa* (groups C–G) is two to three times less than that for clonal lineages (groups A and B, Table 1), suggesting that barriers to genetic exchange between lineages within *M. aeruginosa* could be formed in a DNA sequence homology-dependent manner. However, it should be noted that LD analyses of subgroups within groups A and B indicated significant LD, although the genetic divergence within these subgroups is equivalent to that of freely recombining lineages (e.g., group A1). Given that distantly related individuals are often co-isolated from a single bloom (e.g., [5]), physical isolation between each clonal lineage is likely to be absent. Lineage-specific occurrence of genetic exchange, such as phage-mediated transduction in the presence of different cyanophage susceptibility among lineages [19], is thus likely to be responsible for the heterogeneous impact of recombination between lineages of *M. aeruginosa*. Because the recombination rate is known to decrease log-linearly with increasing genetic distance between individuals [20], [21], the high frequency of recombination observed in multiple intraspecific lineages of an overall clonal species *per se* is not surprising. A similar pattern has been obtained for other bacteria [22], [23] and has been referred to elsewhere as “cryptic sex” [24]. Interestingly, among the five panmictic lineages, two lines of evidence suggest that group G represents a locally expanding lineage.

**Table 1:** Genetic diversity and recombination.

Group	*N* a	*n* b	*S* c	*π* d	*I_A_^S^* e
A	111	50	185	0.0159	0.274*
A1	30	12	54	0.0067	0.191*
A2	33	22	100	0.0106	0.265*
A3	43	14	62	0.0065	0.172*
B	69	54	166	0.0136	0.123*
B1	63	50	158	0.0131	0.140*
C	33	6	61	0.0090	−0.023
D	31	12	38	0.0048	0.056
E	29	13	51	0.0055	0.010
F	11	6	46	0.0066	−0.024
G	70	44	96	0.0076	0.089*
G1	54	40	66	0.0067	0.066*(0.037)^f^
G2	44	24	51	0.0046	0.069
Other	58	52	355	0.0285	0.363*

a Number of isolates.

b Number of ST.

c Number of segregating sites.

d Nucleotide diversity using unique STs.

e Standardized index of association. Only unique STs were used to avoid the effect of biased sample collections. Null hypothesis of panmixia (*I_A_^S^* = 0) is tested against significant linkage disequilibrium (*I_A_^S^*>0). *P<0.001.

f The value in parentheses is based on analysis of using six loci (excluding a highly polymorphic locus *glnA*).

**Figure 2:**
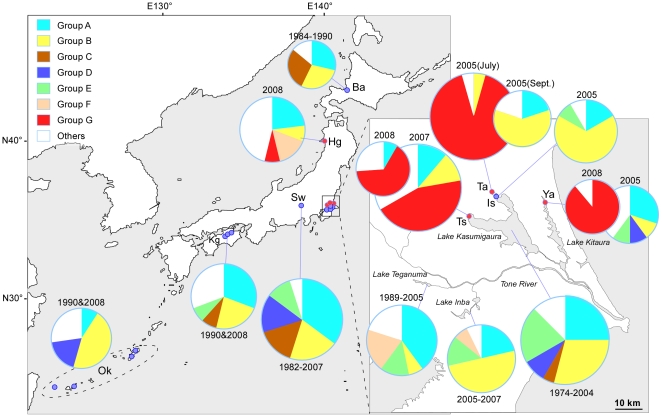
Distribution of *Microcystis aeruginosa* STs in Japan. ST proportions at each sampling location are displayed as pie charts, the size of each being proportional to the number of STs. Intraspecies groups are indicated by different colors as defined in the phylogenetic tree in Fig. 1. Note that the same ST is included in the number of STs in the pie chart indicating pooled samples when they were isolated from different points of time or place. Furthermore, the same ST is not included when it was isolated from the same bloom to avoid the effect of biased sample collections. Red circles on the map indicate locations from where group G strains were isolated whereas blue circles indicate locations from where group G strains were not obtained. Abbreviations for sampling locations are as follows: Ba, Lake Barato; Hg, Hachiro Lagoon; Sw, Lake Suwa; Kg, reservoirs in Kagawa Prefecture; Ok, dams in Okinawa Prefecture; Ta, Takasaki; Is, Iseki; Ts, Tsuchiura; and Ya, Yasuzuka.

**Figure 3:**
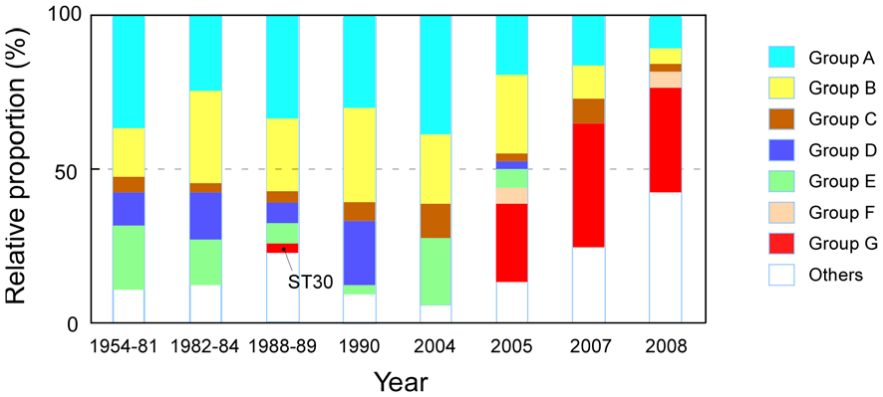
Distribution of the relative proportions of different phylogenetic groups. Strains isolated prior to 1990 were arbitrarily pooled to increase the sample size to include at least 18 STs for each bar representation. Strains isolated in 1996–2000 and 2006 were not included due to the small number of isolates available (eight and three strains, respectively). As in Fig. 2, the same ST is included in the number of STs indicated in bar charts when it had been isolated from different points of time or place and not included when isolated from the same bloom.

First, all group G isolates were recovered from a small geographical area, Lakes Kasumigaura and Kitaura that comprise the continuous water body of the Lake Kasumigaura Basin since 2005 (Fig. 2), despite a substantial number of isolates, including more than 30 isolates from Lake Kasumigaura in 2004 (and earlier), being previously characterized (Fig. 3). Yates corrected chi-squared test demonstrated that groups A–F had been evenly recovered from different time points (*χ^2^* = 78.86, d.f. = 35, *P* = 0.03), confirming that the non-availability of group G prior to 2004 is significant. ST30 is the only exception, having been isolated in Nepal in 1988. Given the large genetic distance between ST30 and other group G strains (Fig. 1), ST30 may represent one of the ancestral genotypes of group G. Another exception is a single isolate belonging to group G (ST227) recovered from the Hachiro Lagoon (Fig. 1). Given that the Lake Kasumigaura Basin and Hachiro Lagoon share the same activity of game fishing, the discovery of a closely related genotype at some distance from Lake Kasumigaura might represent a very recent artificial transfer between these two aquatic environments (e.g., mediated by fish stocking or fishing boats).

Second, group G appears to be highly prevalent in the recent Lake Kasumigaura Basin bloom, i.e., in the presence of group G, strains belonging to other groups have rarely been recovered from the same bloom of *M. aeruginosa*. Indeed, the recent occurrence of water blooms in summer in the Lake Kasumigaura Basin has been mostly attributed to group G and not to strains belonging to other lineages (Fig. 2). For example, 21 out of 23 STs isolated from Lake Kasumigaura, Takasaki in the early summer of 2005 belonged to group G. A similar pattern was obtained for other time points in Lakes Kasumigaura and Kitaura. At first glance, the bloom in Lake Kasumigaura, Tsuchiura in 2007 appeared to have contained a substantial number of STs belonging to other groups as well as to group G. Given that the dramatic temporal transition of genotypes is known to occur in the population of *M. aeruginosa* in this lake [5], we consider that this anomaly is due to the inadequate pooling of isolates at different time points from summer to autumn. As expected, an exclusion of October isolates from this data set demonstrated that 10 out of 14 STs belonged to group G, assuring the predominance of group G in this locality in 2007. What is interesting here is that numerous closely related but distinct genotypes in group G were found in a single bloom in contrast to the previously observed pattern of genetic diversity of *Microcystis* blooms, where multiple, distinct but distantly related genotypes affiliated into different phylogenetic groups co-existed (Fig. 2, [5], [25]). One might think that the observed dominance of group G in a single bloom may be an artifact due to biased samplings favoring the specific detection of group G. Two lines of evidence suggest that this is not the case. First, most strains belonging to group G indicate a unique colony morphology (Fig. S1). Microscopic observation of the bloom associated with group G in the Lake Kasumigaura Basin indicated that majority of *Microcystis* showed a “group G-type” colony morphology, whereas it has never been observed for isolates obtained from other localities, at least in Japan, excluding the possibility of biased isolation. Second, we used the MA medium [26], an optimum medium for *M. aeruginosa* isolation that allowed us to successfully recover more than 90% of isolates, excluding the possible biased culture.

Interestingly, the “population snapshot” of group G illustrated by eBURST (a program that can identify the recent divergence of clones on the basis of allele sharing at most multiple loci) [27] clearly indicated the deficiency of clonal complexes (Fig. S2), probably reflecting some genetic discontinuity among the strains. This suggests that the observed genetic diversity of group G represents a long-term (millions of years of) evolutionary consequence rather than a recent diversification. Thus, the most likely explanation of the emergence of group G is that this group is a cryptic ecotype [28]: an ecologically distinct phylogenetic cluster previously present at a low frequency in the Lake Kasumigaura Basin. In response to recent environmental changes favorable to it, group G strains may have dramatically increased in numbers to become a major constituent of water blooms in summer in the Lake Kasumigaura Basin. Indeed, the result of Ecotype Simulation [29] identified groups G1 and G2 as putative ecotypes (Fig. 1). In such a case, there may be further ecological specialization within group G. Interestingly, Group G strains show distinct colony morphology (Fig. S1) that may be beneficial under favorable environmental conditions. Alternatively, the occurrence of group G blooms might have resulted from their recent introduction into Lake Kasumigaura from previously unexplored sources. However, we consider this less probable because a recent introduction of numerous closely related genotypes to a small area is unrealistic and has never been observed for other lineages of *M. aeruginosa* or documented for other free-living microbes. Taken together, our results suggest that group G represents an expanding lineage in a small geographical area within the past decade. Although a number of cases of a local expansion of pathogenic bacteria have been documented (e.g., [30]), to our knowledge, this is the first report of local expansion of a within-species phylogenetic cluster for aquatic microbes.

Furthermore, frequent recombination within group G is highly suggestive with regard to bacterial population genetics. The emergence of highly adaptive or invasive bacterial clones is often explained by invoking the “epidemic” population structure in which a small number of successful clones dramatically increase their population size against the few background genotypes [8]. Despite the superficial resemblance of the group G-associated bloom to epidemics, the population structure of this bloom is markedly different from that of epidemics. This bloom contains multiple, closely related group G genotypes between which recombination is frequent and a small number of genotypes belonging to different groups between which recombination is rarely observed.

In this study, we have identified multiple panmictic lineages within *M. aeruginosa*. Because recombination can function as a cohesive force to limit the divergence of each lineage, the single species *M. aeruginosa* can be recognized as an assemblage of multiple, independent phylogenetic units whose evolutionary consequence could also be distinct. Among these, group G is notable for both basic evolutionary interests and local environmental concerns because it is predominantly responsible for the recent occurrence of water blooms in a restricted region in Japan, although all group G isolates are fortunately nontoxic (Tanabe et al., unpublished data). Although available data suggests that group G is a possible adaptive ecotype, caution is required because a sequence-based cluster can be formed in the absence of ecological selection where the frequency of recombination is negatively correlated to DNA sequence divergence [31]–[33], which is the case for *M. aeruginosa* including group G. Furthermore, a possibility that group G was formed as a result of geographic isolation and neutral genetic drift exists. Endemism has long been considered unusual for free-living microbes [34], until possible endemic clades were reported for cyanobacteria [35]–[37]. Because our data are largely limited to Japanese isolates, culture-independent methods specifically to detect group G strains from blooms worldwide would be of great help in dealing with this issue. In any event, ecological characterization is mandatory to confirm the ecotypic status of group G. Long-term examination of genetic diversity of group G and other groups would provide us with a unique opportunity to investigate the evolutionary consequence of this lineage in light of various bacterial speciation theories [38]–[39].

## Materials and Methods

### Strains

A total of 412 strains of *Microcystis aeruginosa* representing 237 STs were used in this study (Table S1). These included 268 isolates (representing 155 STs) that were previously characterized [4]–[6], 12 strains obtained from MCC-NIES (Tsukuba, Japan) representing seven novel STs, and 131 strains that we had isolated from several locations in Lakes Kasumigaura and Kitaura as well as from other localities across Japan and South Asia during 2004–2008, representing 74 novel STs. In addition, an *in silico* survey of MLST loci within the published contigs of *M. aeruginosa* PCC7806 [40] indicated that this strain represents a unique ST (designated ST237).

### Multi-locus sequence typing (MLST)

Isolation and establishment of clone cultures were performed following a protocol previously described [5], [6]. Most strains were established from a single bacterial cell. A few strains were established from a single small colony (consisting of two to five densely aggregated cells enveloped in gelatinous material), but sequence analysis confirmed that these are represented by a single genotype (i.e., clonal).

MLST was performed following a previously described protocol [4]–[6]. Sequence data have been deposited in the DDBJ database under accession numbers AB547713–AB547902. For each locus, each allele was assigned a different arbitrary number and a unique combination of seven allele numbers (allelic profile) was defined as a strain's ST. No indels were found within the other six loci, whereas an insertion of 3 bp was found within allele 89 at *tpi* in ST226 near its 3′ end. This insertion was excluded for phylogenetic analysis.

### Phylogenetic and population genetic analyses

Neighbor-joining (NJ) phylogenetic tree of 237 STs of *M. aeruginosa* was constructed on the basis of the distance matrix calculated using the concatenated sequence of the seven MLST loci, employing the maximum composite likelihood substitution model using MEGA software ver. 4.01 [41]. Bootstrap statistical support was estimated from 1000 resamplings of data. Nucleotide diversity [42] using unique STs, was calculated by DnaSP ver. 4. 00 [43]. Standardized index of association calculated using START ver. 2 [44]. Bayesian assignment analysis, a method for dividing a collection of isolates into multiple genetic clusters on the basis of difference in allelic frequency at multiple marker loci was performed using BAPS ver. 5.2 [16] on the basis of the “Codon linkage model,” employing the option of “clustering with linked loci.” We determined eight populations that were most appropriate to explain the data. Using the same software, population admixture analysis was performed on the basis of the results obtained from Bayesian assignment analysis with the default setting, except that the number of iterations for the estimation of admixture coefficients was set at 100 and the number of reference individuals from each population was set at 200, as suggested by the authors (see the BAPS manual). Ecotype Simulation [29] was performed with 191 STs using default settings. With the exception of ST40, which was used for an outgroup, STs belonging to neither groups A–G nor X were included due to computational limitation (as suggested by the authors). Estimated parameter values were shown in Table S3.

## Supporting Information

Figure S1 **Colony morphology of group G strains in field water samples.** A small number of relatively large cells (5.5–7.5 µm) loosely aggregated to form irregular small colonies, but never formed the large sponge-like structure that was previously identified as *Microcystis aeruginosa* (sensu Komárek, 1991). In culture, however, we observed that it sometimes formed larger colonies. Reference: Komárek, J. (1991). A review of water-bloom forming *Microcystis* species, with regard to populations from Japan. Arch Hydrobiol Suppl Algol Stud 64: 115–127.
(PPT)

Figure S2 **Population snapshot of** ***Microcystis aeruginosa*** **group G.** eBURST ver. 3 (available at http://eburst.mlst.net/default.asp) [27] was used to illustrate the snapshot. Each circle indicates a distinct ST accompanied by the corresponding number. Circle size is proportional to the abundance of isolates of that ST. Pairs of circles, which together represent a single locus variant (SLV; the ST differs at only one of the seven alleles), are connected by a line. The group of STs connected by lines form a “clonal complex.” The red circle (ST116) indicates a putative founder genotype of the clonal complex. To avoid the effect of biased isolation of strains, only a single ST was included to represent more than two strains isolated from the same place and time. Note that analysis without the highly polymorphic locus *glnA* gives essentially the same picture (data not shown).
(PPT)

Table S1 **Strains of** ***Microcystis aeruginosa*** **used in this study, their location, date of collection and details.**
(DOC)

Table S2 **Genetic diversity and recombination.** The results of maximum likelihood tests for tree congruence proposed by Feil *et al.* [9] supported the recombinogenic nature of the five lineages, where we observed a higher proportion of phylogenetic congruence between the phylogenetic tree of each locus and randomized ones. The estimated parameter values for relative impact (*r/m*) and rate (*ρ/θ*) of recombination and mutation also indicated the higher impact of recombination groups D and G1, where we observed higher values than for other lineages. On the other hand, both parameter estimates for groups C and F were less than 1, suggesting the lower frequency of recombination relative to point mutation. On the basis of the results obtained from analysis using the entire data set, we employed a constant value for the mean tract length of imported gene segments (*δ* = 225 bps) to infer the recombination parameters for each lineage. Because this value is much smaller than the length of all MLST loci (>400 bps), we probably failed to capture some of the entire-allele recombinational replacements. Thus, the inconsistency between the results of the LD analysis and recombination parameter inference could be due to the underestimation of the relative rate of recombination. Reference: Didelot X, Falush D (2007) Inference of bacterial microevolution using multilocus sequence data. Genetics 175: 1251–1266. Swofford DL (2002) PAUP* – Phylogenetic analysis using parsimony (*and other methods), version 4. Sunderland, MA: Sianuer Associates. Tajima F (1989) Statistical method for testing the neutral mutation hypothesis by DNA polymorphism. Genetics 123: 585–595.
(DOC)

Table S3 **Result of Ecotype Simulation.** The values in parenthesis indicate 95% confidence intervals for each parameter estimated. Note that ecotype demarcation analysis implemented in Ecotype Simulation software conservatively identified 85 putative ecotypes, which was lesser than that estimated by simulation analysis. However, the result indicated many more ecotypes than expected. Nevertheless, groups G1 and G2 without G1 were predicted as ecotypes (see also Fig. 1).
(DOC)

## References

[1] Carmichael WW, Watanabe MF, Harada K, Carmichael WW, Fujiki H (1996). Toxic *Microcystis* and the environment.. Toxic *Microcystis*.

[2] Beasley VR, Cook WO, Dahlem AM, Hooser SB, Lovell RA (1989). Algae intoxication in livestock and waterfowl.. Food Anim Pract.

[3] Jochimsen EM, Carmichael WW, An JS, Cardo DM, Cookson ST (1998). Liver failure and death after exposure to microcystins at a hemodialysis center in Brazil.. N Engl J Med.

[4] Tanabe Y, Kasai F, Watanabe MM (2007). Multilocus sequence typing (MLST) reveals high genetic diversity and clonal population structure of the toxic cyanobacterium *Microcystis aeruginosa*.. Microbiology.

[5] Tanabe Y, Kasai F, Watanabe MM (2009). Fine-scale spatial and temporal genetic differentiation of water bloom-forming cyanobacterium *Microcystis aeruginosa*: revealed by multilocus sequence typing.. Environ Microbiol Rep.

[6] Tanabe Y, Sano T, Kasai F, Watanabe MM (2009). Recombination, cryptic clades and neutral molecular divergence of the microcystin synthetase (*mcy*) genes of toxic cyanobacterium *Microcystis aeruginosa*.. BMC Evol Biol.

[7] Spratt BG, Hanage WP, Feil EJ (2001). The relative contributions of recombination and point mutation to the diversification of bacterial clones.. Curr Opin Microbiol.

[8] Smith JM, Smith NH, O'Rourke M, Spratt BG (1993). How clonal are bacteria?. Proc Natl Acad Sci U S A.

[9] Feil EJ, Holmes EC, Bessen DE, Chan MS, Day NP (2001). Recombination within natural populations of pathogenic bacteria: short-term empirical estimates and long-term phylogenetic consequences.. Proc Natl Acad Sci U S A.

[10] Pérez-Losada M, Browne EB, Madsen A, Wirth T, Viscidi RP (2006). Population genetics of microbial pathogens estimated from multilocus sequence typing (MLST) data.. Infect Genet Evol.

[11] Tanabe Y, Kaya K, Watanabe MM (2004). Evidence for recombination in the microcystin synthetase (*mcy*) genes of toxic cyanobacteria *Microcystis* spp.. J Mol Evol.

[12] Kurmayer R, Christiansen G, Gumpenberger M, Fastner J (2005). Genetic identification of microcystin ecotypes in toxic cyanobacteria of the genus *Planktothrix*.. Microbiology.

[13] Fewer DP, Rouhiainen L, Jokela J, Wahlsten M, Laakso K (2007). Recurrent adenylation domain replacement in the microcystin synthetase gene cluster.. BMC Evol Biol.

[14] Lodders N, Stackebrandt E, Nübel U (2005). Frequent genetic recombination in natural populations of the marine cyanobacterium *Microcoleus chthonoplastes*.. Environ Microbiol.

[15] Barker GL, Handley BA, Vacharapiyasophon P, Stevens JR, Hayes PK (2000). Allele-specific PCR shows that genetic exchange occurs among genetically diverse *Nodularia* (cyanobacteria) filaments in the Baltic Sea.. Microbiology.

[16] Corander J, Marttinen P, Sirén J, Tang J (2008). Enhanced Bayesian modelling in BAPS software for learning genetic structures of populations.. BMC Bioinformatics.

[17] Lawrence JG (2002). Gene transfer in bacteria: speciation without species?. Theor Popul Biol.

[18] Haubold B, Hudson RR (2000). LIAN 3.0: detecting linkage disequilibrium in multilocus data. Linkage Analysis.. Bioinformatics.

[19] Deng L, Hayes PK (2008). Evidence for cyanophages active against bloom-forming freshwater cyanobacteria.. Freshwater Biol.

[20] Vulić M, Dionisio F, Taddei F, Radman M (1997). Molecular keys to speciation: DNA polymorphism and the control of genetic exchange in enterobacteria.. Proc Natl Acad Sci U S A.

[21] Majewski J, Zawadzki P, Pickerill P, Cohan FM, Dowson CG (2000). Barriers to genetic exchange between bacterial species: *Streptococcus pneumoniae* transformation.. J Bacteriol.

[22] Goss EM, Kreitman M, Bergelson J (2005). Genetic diversity, recombination and cryptic clades in *Pseudomonas viridiflava* infecting natural populations of *Arabidopsis thaliana*.. Genetics.

[23] den Bakker HC, Didelot X, Fortes ED, Nightingale KK, Wiedmann M (2008). Lineage specific recombination rates and microevolution in *Listeria monocytogenes*.. BMC Evol Biol.

[24] Souza V, Nguyen TT, Hudson RR, Piñero D, Lenski RE (1992). Hierarchical analysis of linkage disequilibrium in *Rhizobium* populations: evidence for sex?. Proc Natl Acad Sci U S A.

[25] Janse I, Kardinaal WE, Meima M, Fastner J, Visser PM (2004). Toxic and nontoxic *Microcystis* colonies in natural populations can be differentiated on the basis of rRNA gene internal transcribed spacer diversity.. Appl Environ Microbiol.

[26] Kasai F, Kawachi M, Erata M, Watanabe MM (2004). NIES-Collection, List of strains, microalgae and protozoa, 7th edn.

[27] Feil EJ, Li BC, Aanensen DM, Hanage WP, Spratt BG (2004). eBURST: inferring patterns of evolutionary descent among clusters of related bacterial genotypes from multilocus sequence typing data.. J Bacteriol.

[28] Cohan FM (2002). What are bacterial species?. Annu Rev Microbiol.

[29] Koeppel A, Perry EB, Sikorski J, Krizanc D, Warner WA (2008). Identifying the fundamental units of bacterial diversity: a paradigm shift to incorporate ecology into bacterial systematics.. Proc Natl Acad Sci U S A.

[30] Hoen AG, Margos G, Bent SJ, Diuk-Wasser MA, Barbour A (2009). Phylogeography of *Borrelia burgdorferi* in the eastern United States reflects multiple independent Lyme disease emergence events.. Proc Natl Acad Sci U S A.

[31] Falush D, Torpdahl M, Didelot X, Conrad DF, Wilson DJ (2006). Mismatch induced speciation in *Salmonella*: model and data.. Philos Trans R Soc Lond B Biol Sci.

[32] Hanage WP, Spratt BG, Turner KM, Fraser C (2006). Modelling bacterial speciation.. Philos Trans R Soc Lond B Biol Sci.

[33] Fraser C, Hanage WP, Spratt BG (2007). Recombination and the nature of bacterial speciation.. Science.

[34] Baas Becking LGM (1934). Geobiologie of inleiding tot de milieukunde.

[35] Papke RT, Ramsing NB, Bateson MM, Ward DM (2003). Geographical isolation in hot spring cyanobacteria.. Environ Microbiol.

[36] Hongmei J, Aitchison JC, Lacap DC, Peerapornpisal Y, Sompong U (2005). Community phylogenetic analysis of moderately thermophilic cyanobacterial mats from China, the Philippines and Thailand.. Extremophiles.

[37] Ionescu D, Hindiyeh M, Malkawi H, Oren A (2010). Biogeography of thermophilic cyanobacteria: insights from the Zerka Ma'in hot springs (Jordan).. FEMS Microbiol Ecol.

[38] Gevers D, Cohan FM, Lawrence JG, Spratt BG, Coenye T (2005). Re-evaluating prokaryotic species.. Nat Rev Microbiol.

[39] Fraser C, Alm EJ, Polz MF, Spratt BG, Hanage WP (2009). The bacterial species challenge: making sense of genetic and ecological diversity.. Science.

[40] Frangeul L, Quillardet P, Castets AM, Humbert JF, Matthijs HC (2008). Highly plastic genome of *Microcystis aeruginosa* PCC 7806, a ubiquitous toxic freshwater cyanobacterium.. BMC Genomics.

[41] Tamura K, Dudley J, Nei M, Kumar S (2007). MEGA4: Molecular Evolutionary Genetics Analysis (MEGA) software version 4.0.. Mol Biol Evol.

[42] Nei M (1987). Molecular Evolutionary Genetics.

[43] Rozas J, Sanchez-DelBarrio JC, Messeguer X, Rozas R (2003). DnaSP, DNA polymorphism analyses by the coalescent and other methods.. Bioinformatics.

[44] Jolley KA, Feil EJ, Chan MS, Maiden MC (2001). Sequence type analysis and recombinational tests (START).. Bioinformatics.

[45] Kaneko T, Nakajima N, Okamoto S, Suzuki I, Tanabe Y (2007). Complete genomic structure of the bloom-forming toxic cyanobacterium *Microcystis aeruginosa* NIES-843.. DNA Res.

