# Supplementary material for: Local Expansion of a Panmictic Lineage of Water Bloom-Forming Cyanobacterium Microcystis aeruginosa
Source: PLoS One. 2011 Feb 24;6(2):e17085. doi: 10.1371/journal.pone.0017085 (PMC3044731; doi:10.1371/journal.pone.0017085)
Supplement: Figure S1 — Colony morphology of group G strains in field water samples. A small number of relatively large cells (5.5–7.5 µm) loosely aggregated to form irregular small colonies, but never formed the large sponge-like structure that was previously identified as Microcystis aeruginosa (sensu Komárek, 1991). In culture, however, we observed that it sometimes formed larger colonies. Reference: Komárek, J. (1991). A review of water-bloom forming Microcystis species, with regard to populations from Japan. Arch Hydrobiol Suppl Algol Stud 64: 115–127. (PPT) [file pone.0017085.s001.ppt]

## Slide 1
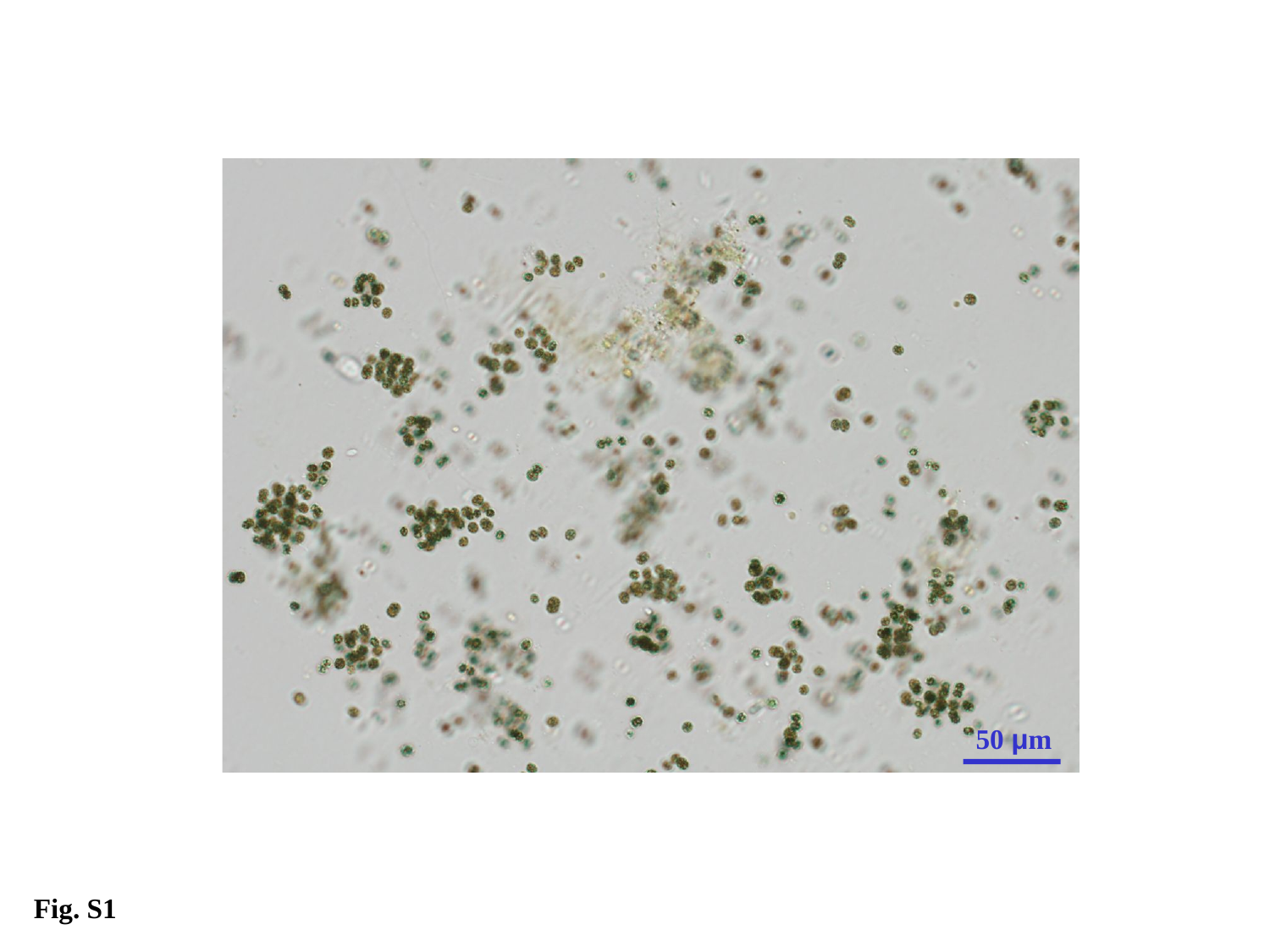

50 μm
Fig. S1

## Slide 2
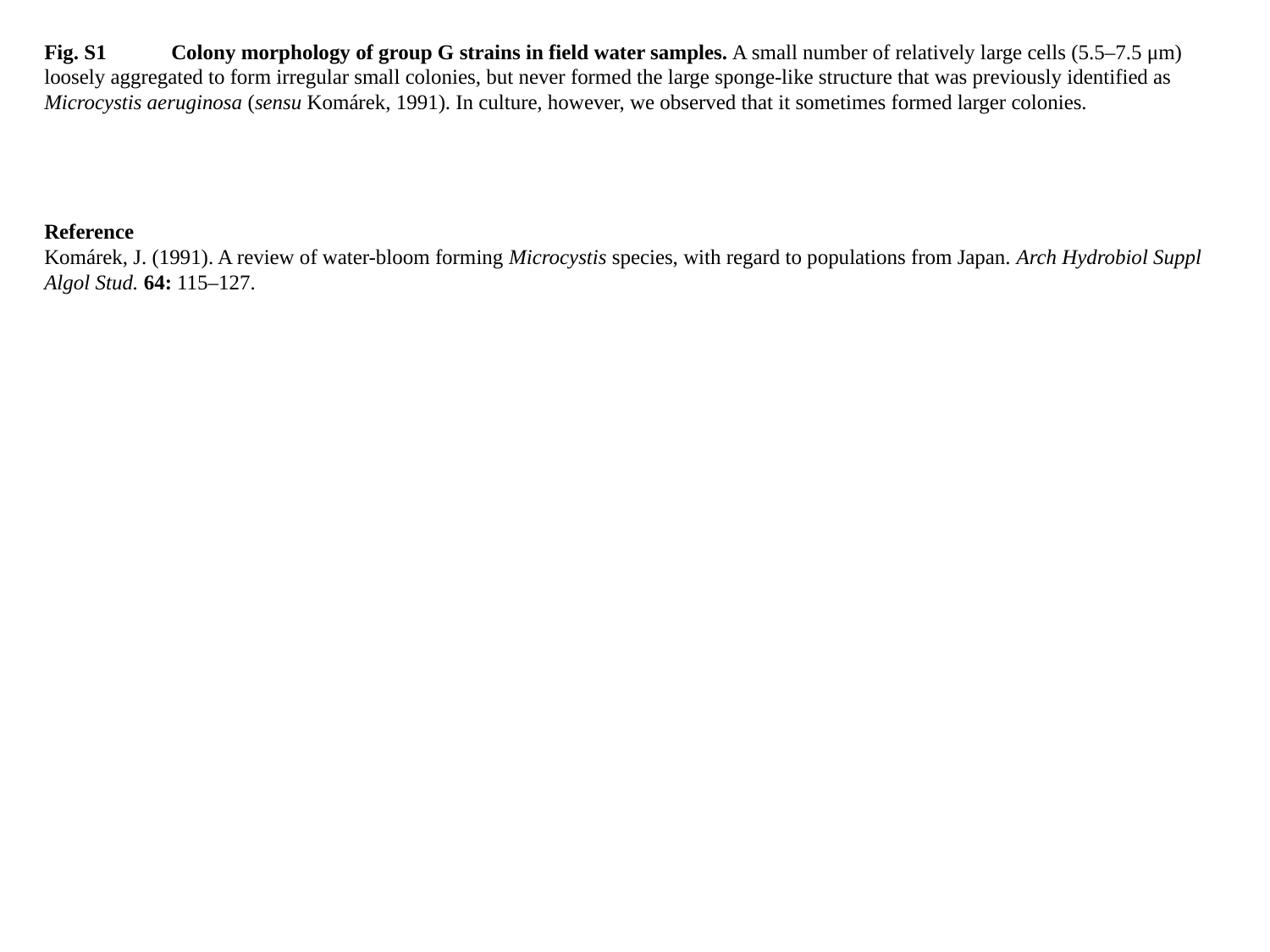

Fig. S1	Colony morphology of group G strains in field water samples. A small number of relatively large cells (5.5–7.5 μm) loosely aggregated to form irregular small colonies, but never formed the large sponge-like structure that was previously identified as Microcystis aeruginosa (sensu Komárek, 1991). In culture, however, we observed that it sometimes formed larger colonies.
Reference
Komárek, J. (1991). A review of water-bloom forming Microcystis species, with regard to populations from Japan. Arch Hydrobiol Suppl Algol Stud. 64: 115–127.
