# Supplementary material for: Local Expansion of a Panmictic Lineage of Water Bloom-Forming Cyanobacterium Microcystis aeruginosa
Source: PLoS One. 2011 Feb 24;6(2):e17085. doi: 10.1371/journal.pone.0017085 (PMC3044731; doi:10.1371/journal.pone.0017085)
Supplement: Figure S2 — Population snapshot of Microcystis aeruginosa group G. eBURST ver. 3 (available at http://eburst.mlst.net/default.asp) [27] was used to illustrate the snapshot. Each circle indicates a distinct ST accompanied by the corresponding number. Circle size is proportional to the abundance of isolates of that ST. Pairs of circles, which together represent a single locus variant (SLV; the ST differs at only one of the seven alleles), are connected by a line. The group of STs connected by lines form a “clonal complex.” The red circle (ST116) indicates a putative founder genotype of the clonal complex. To avoid the effect of biased isolation of strains, only a single ST was included to represent more than two strains isolated from the same place and time. Note that analysis without the highly polymorphic locus glnA gives essentially the same picture (data not shown). (PPT) [file pone.0017085.s002.ppt]

## Slide 1
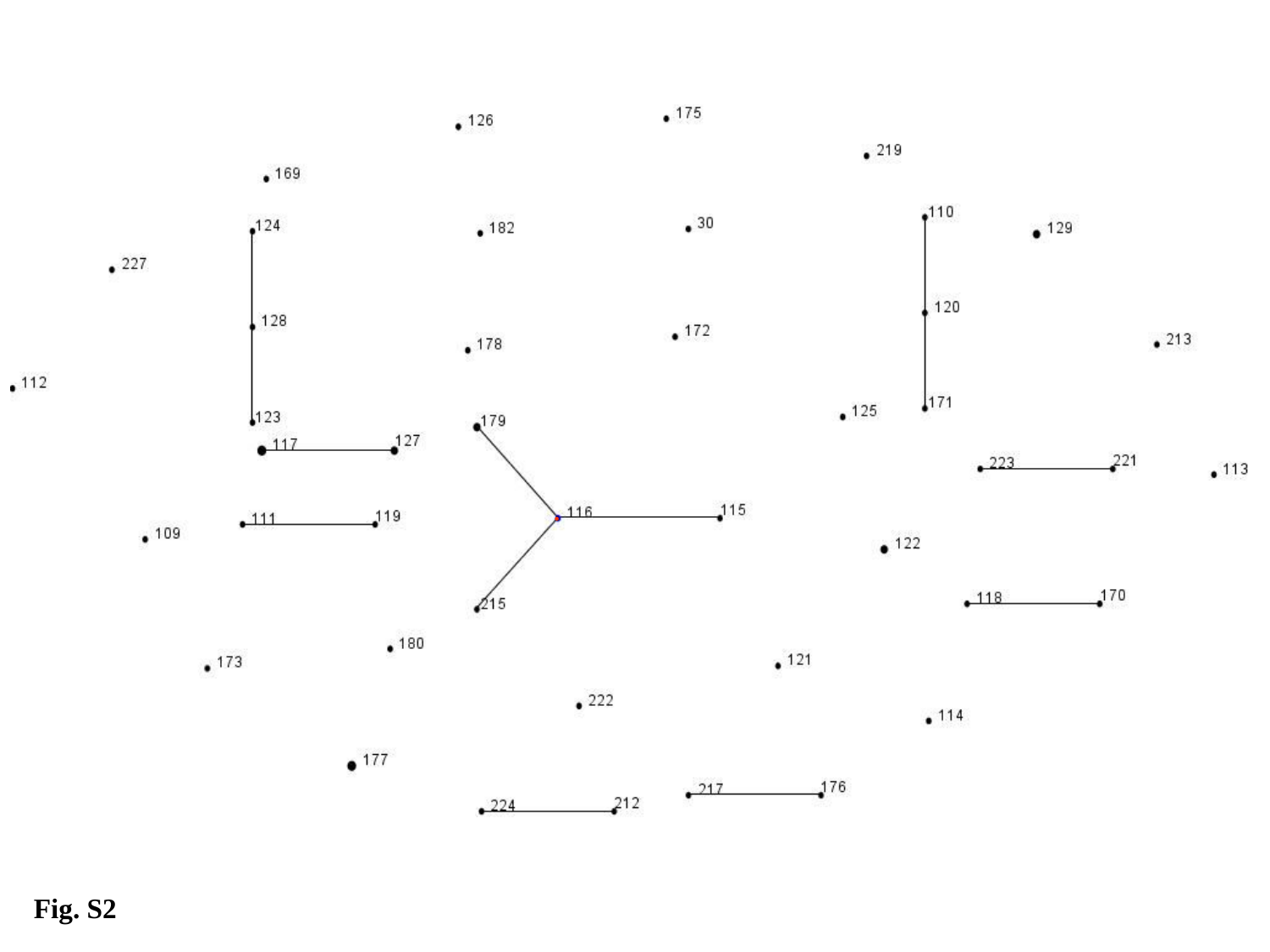

●
Fig. S2

## Slide 2
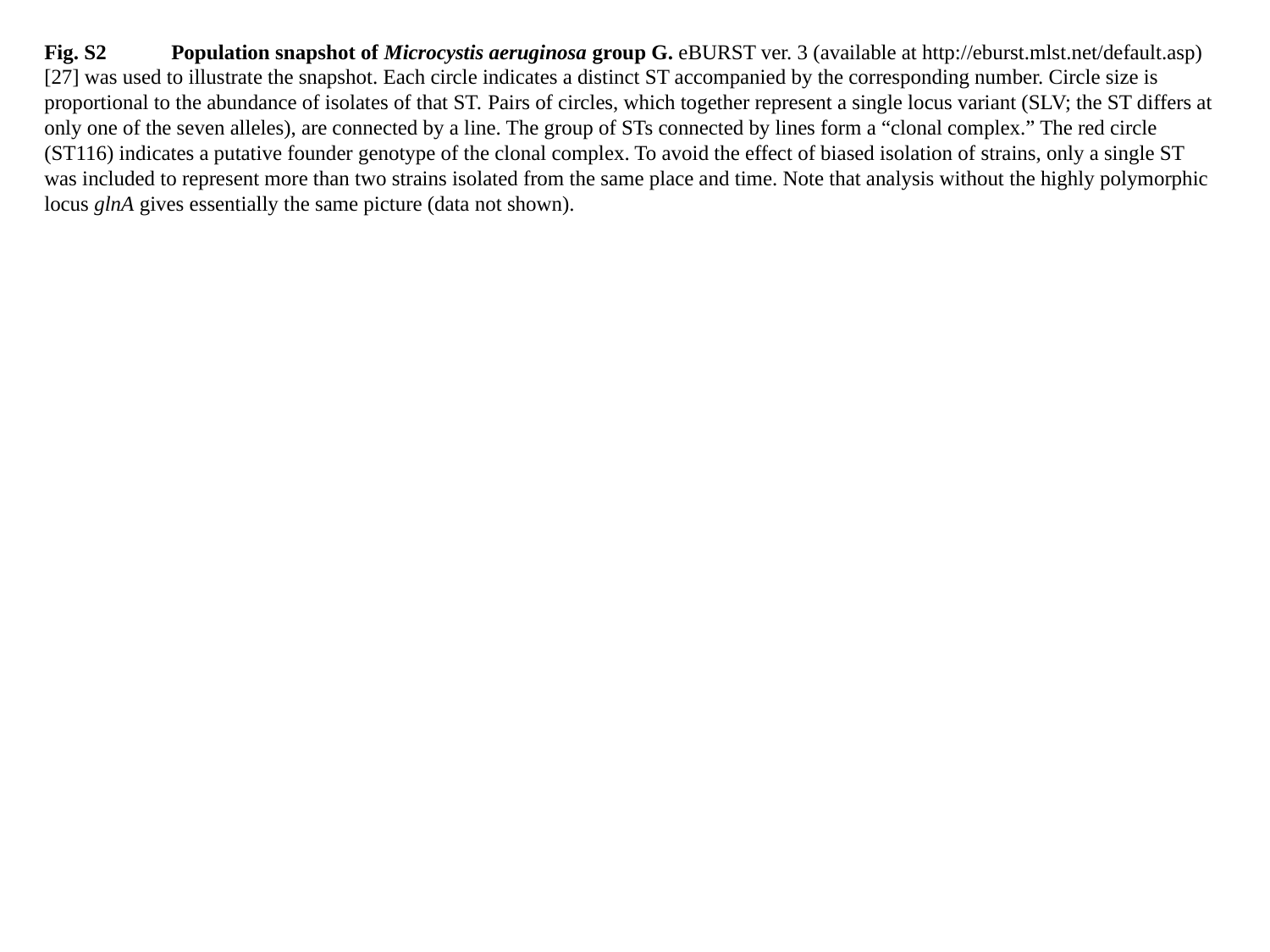

Fig. S2	Population snapshot of Microcystis aeruginosa group G. eBURST ver. 3 (available at http://eburst.mlst.net/default.asp) [27] was used to illustrate the snapshot. Each circle indicates a distinct ST accompanied by the corresponding number. Circle size is proportional to the abundance of isolates of that ST. Pairs of circles, which together represent a single locus variant (SLV; the ST differs at only one of the seven alleles), are connected by a line. The group of STs connected by lines form a “clonal complex.” The red circle (ST116) indicates a putative founder genotype of the clonal complex. To avoid the effect of biased isolation of strains, only a single ST was included to represent more than two strains isolated from the same place and time. Note that analysis without the highly polymorphic locus glnA gives essentially the same picture (data not shown).
