# Supplementary material for: Local Expansion of a Panmictic Lineage of Water Bloom-Forming Cyanobacterium Microcystis aeruginosa
Source: PLoS One. 2011 Feb 24;6(2):e17085. doi: 10.1371/journal.pone.0017085 (PMC3044731; doi:10.1371/journal.pone.0017085)
Supplement: Table S1 — Strains of Microcystis aeruginosa used in this study, their location, date of collection and details. (DOC) [file pone.0017085.s003.doc]

**Table S1.** Strains of *Microcystis aeruginosa* used in this study, their location, date of collection and details.

| **Strain** | **Locality**^a^ | **Date**^b^ | **ST^c^** | **Allele numbers** | | | | | | | **Group** |
| --- | --- | --- | --- | --- | --- | --- | --- | --- | --- | --- | --- |
|  |  |  |  | ***ftsZ*** | ***glnA*** | ***gltX*** | ***gyrB*** | ***pgi*** | ***recA*** | ***tpi*** |  |
| PCC7941 | Lake Little Lideau, Canada | */*/54 | **21** | 14 | 17 | 17 | 14 | 18 | 6 | 15 | B |
| PCC7806 | Braakman reservoir, Netherlands | */*/72 | **237** | 20 | 128 | 133 | 98 | 111 | 101 | 91 | NA |
| NIES44 | Lake Kasumigaura | */8/74 | **1** | 1 | 1 | 1 | 1 | 1 | 1 | 1 | E |
| NIES478 | Lake Kasumigaura | */9/77 | **62** | 14 | 17 | 44 | 36 | 43 | 40 | 6 | B |
| NIES604 | Lake Kasumigaura | */9/77 | **17** | 12 | 14 | 14 | 11 | 15 | 13 | 12 | C |
| TAC4 | Lake Kasumigaura | */1/78 | **34** | 21 | 26 | 27 | 21 | 26 | 22 | 23 | NA |
| TAC6 | Lake Kasumigaura | */1/78 | **34** | 21 | 26 | 27 | 21 | 26 | 22 | 23 | NA |
| TAC15 | Lake Kasumigaura | */2/78 | **24** | 7 | 8 | 20 | 7 | 21 | 16 | 18 | D |
| TAC19 | Lake Kasumigaura | */2/78 | **25** | 7 | 8 | 20 | 7 | 21 | 17 | 18 | D |
| TAC20 | Lake Kasumigaura | */2/78 | **24** | 7 | 8 | 20 | 7 | 21 | 16 | 18 | D |
| NIES110 | Lake Kasumigaura | */8/78 | **1** | 1 | 1 | 1 | 1 | 1 | 1 | 1 | E |
| NIES111 | Lake Kasumigaura | */8/78 | **14** | 11 | 12 | 11 | 1 | 12 | 12 | 1 | E |
| TAC38 | Lake Kasumigaura | */8/78 | **26** | 9 | 15 | 21 | 12 | 16 | 8 | 8 | A |
| TAC39 | Lake Kasumigaura | */8/78 | **1** | 1 | 1 | 1 | 1 | 1 | 1 | 1 | E |
| TAC40 | Lake Kasumigaura | */8/78 | **1** | 1 | 1 | 1 | 1 | 1 | 1 | 1 | E |
| NIES103 | Lake Kasumigaura | */12/78 | **26** | 9 | 15 | 21 | 12 | 16 | 8 | 8 | A |
| TAC45 | Lake Kasumigaura | */12/78 | **26** | 9 | 15 | 21 | 12 | 16 | 8 | 8 | A |
| TAC46 | Lake Kasumigaura | */12/78 | **26** | 9 | 15 | 21 | 12 | 16 | 8 | 8 | A |
| NIES299 | Lake Kasumigaura | */8/79 | **61** | 20 | 45 | 43 | 36 | 33 | 39 | 6 | B |
| NIES88 | Lake Kawaguchi | */6/81 | **3** | 3 | 3 | 3 | 3 | 3 | 3 | 3 | A |
| NIES89 | Lake Kawaguchi | */6/81 | **4** | 4 | 4 | 4 | 4 | 4 | 4 | 4 | A |
| NIES90 | Lake Kawaguchi | */6/81 | **5** | 5 | 5 | 5 | 5 | 5 | 5 | 5 | A |
| NIES107 | Lake Kawaguchi | */6/81 | **13** | 5 | 5 | 5 | 10 | 5 | 11 | 10 | A |
| NIES109 | Lake Yogo | */7/82 | **15** | 12 | 13 | 12 | 11 | 13 | 13 | 11 | C |
| NIES100 | Lake Suwa | */8/82 | **8** | 7 | 8 | 7 | 7 | 7 | 7 | 7 | D |
| NIES108 | Lake Suwa | */8/82 | **14** | 11 | 12 | 11 | 1 | 12 | 12 | 1 | E |
| NIES99 | Lake Suwa | */8/82 | **7** | 6 | 7 | 6 | 6 | 6 | 6 | 6 | B |
| TAC50 | Lake Suwa | 4/10/82 | **9** | 8 | 7 | 6 | 6 | 8 | 6 | 6 | B |
| TAC51 | Lake Suwa | 4/10/82 | **9** | 8 | 7 | 6 | 6 | 8 | 6 | 6 | B |
| TAC57 | Lake Suwa | 4/10/82 | **14** | 11 | 12 | 11 | 1 | 12 | 12 | 1 | E |
| NIES101 | Lake Suwa | */10/82 | **9** | 8 | 7 | 6 | 6 | 8 | 6 | 6 | B |
| NIES112 | Lake Suwa | */10/82 | **16** | 11 | 12 | 13 | 1 | 14 | 1 | 1 | E |
| NIES106 | Lake Kasumigaura | */9/82 | **1** | 1 | 1 | 1 | 1 | 1 | 1 | 1 | E |
| NIES87 | Lake Kasumigaura | */9/82 | **2** | 2 | 2 | 2 | 2 | 2 | 2 | 2 | NA |
| NIES98 | Lake Kasumigaura | */9/82 | **6** | 6 | 6 | 6 | 6 | 6 | 6 | 6 | B |
| NIES102 | Lake Kasumigaura | */9/82 | **10** | 9 | 9 | 8 | 8 | 9 | 8 | 8 | A |
| NIES105 | Lake Kasumigaura | */9/82 | **12** | 11 | 11 | 10 | 1 | 11 | 10 | 1 | E |
| NIES298 | Lake Kasumigaura | */9/82 | **60** | 6 | 44 | 42 | 35 | 42 | 23 | 6 | B |
| NIES91 | Lake Kasumigaura | */9/82 | **77** | 20 | 7 | 55 | 6 | 50 | 6 | 6 | B |
| TAC71 | Hakui, Ishikawa | 10/9/82 | **4** | 4 | 4 | 4 | 4 | 4 | 4 | 4 | A |
| TAC62 | Lake Kutsuzawa, Nagano | 12/9/82 | **4** | 4 | 4 | 4 | 4 | 4 | 4 | 4 | A |
| TAC63 | Lake Kutsuzawa, Nagano | 12/9/82 | **4** | 4 | 4 | 4 | 4 | 4 | 4 | 4 | A |
| TAC65 | Chisato-ike pond, Nagano | 12/9/82 | **27** | 7 | 8 | 22 | 7 | 22 | 16 | 19 | D |
| TAC66 | Rokusuk-ike pond, Nagano | 12/9/82 | **27** | 7 | 8 | 22 | 7 | 22 | 16 | 19 | D |
| TAC67 | Rokusuk-ike pond, Nagano | 12/9/82 | **35** | 22 | 27 | 28 | 20 | 8 | 23 | 6 | B |
| TAC69 | Rokusuk-ike pond, Nagano | 12/9/82 | **36** | 6 | 28 | 28 | 20 | 6 | 23 | 24 | B |
| TAC60 | Lake Shirakaba | 13/9/82 | **29** | 18 | 21 | 3 | 17 | 3 | 14 | 21 | A |
| TAC61 | Lake Shirakaba | 13/9/82 | **29** | 18 | 21 | 3 | 17 | 3 | 14 | 21 | A |
| NIES104 | Imperial Palace, Tokyo | */11/82 | **11** | 10 | 10 | 9 | 9 | 10 | 9 | 9 | NA |
| TAC91 | Lake Barato | 24/8/84 | **2** | 2 | 2 | 2 | 2 | 2 | 2 | 2 | NA |
| TAC92 | Lake Barato | 24/8/84 | **26** | 9 | 15 | 21 | 12 | 16 | 8 | 8 | A |
| TAC93 | Lake Barato | 24/8/84 | **26** | 9 | 15 | 21 | 12 | 16 | 8 | 8 | A |
| TAC95 | Lake Barato | 24/8/84 | **65** | 35 | 23 | 25 | 38 | 44 | 41 | 25 | B |
| TAC86 | Koshi-ike Pond, Shimane | 6/9/84 | **28** | 17 | 20 | 23 | 16 | 23 | 18 | 20 | A |
| TAC96 | Lake Shirakaba | 13/9/84 | **39** | 7 | 8 | 30 | 7 | 29 | 17 | 26 | D |
| TAC97 | Lake Shirakaba | 13/9/84 | **29** | 18 | 21 | 3 | 17 | 3 | 14 | 21 | A |
| TAC98 | Lake Shirakaba | 13/9/84 | **39** | 7 | 8 | 30 | 7 | 29 | 17 | 26 | D |
| TAC74 | Lake Yogo | 9/9/84 | **63** | 20 | 46 | 45 | 37 | 33 | 24 | 6 | B |
| TAC75 | Lake Yogo | 9/9/84 | **64** | 7 | 8 | 22 | 7 | 22 | 17 | 40 | D |
| TAC76 | Lake Yogo | 9/9/84 | **37** | 23 | 29 | 29 | 22 | 27 | 24 | 25 | B |
| TAC109 | Shigure Dam, Ogasawara | */11/84 | **28** | 17 | 20 | 23 | 16 | 23 | 18 | 20 | A |
| TAC110 | Shigure Dam, Ogasawara | */11/84 | **38** | 2 | 2 | 2 | 2 | 28 | 2 | 2 | NA |
| TAC114 | Kathmandu, Nepal | 17/11/88 | **30** | 19 | 22 | 24 | 18 | 24 | 19 | 22 | G |
| TAC115 | Kathmandu, Nepal | 17/11/88 | **40** | 24 | 30 | 31 | 23 | 30 | 25 | 27 | NA |
| TAC134 | Lake Suwa | 24/7/89 | **51** | 20 | 38 | 38 | 20 | 36 | 20 | 35 | B |
| TAC135 | Lake Suwa | 24/7/89 | **52** | 5 | 5 | 5 | 10 | 5 | 11 | 36 | A |
| TAC122 | Lake Barato | 2/8/89 | **66** | 9 | 47 | 8 | 12 | 9 | 8 | 8 | A |
| TAC123 | Lake Barato | 2/8/89 | **66** | 9 | 47 | 8 | 12 | 9 | 8 | 8 | A |
| TAC124 | Lake Barato | 2/8/89 | **67** | 12 | 48 | 46 | 11 | 15 | 42 | 41 | C |
| TAC125 | Lake Barato | 2/8/89 | **31** | 20 | 23 | 25 | 19 | 25 | 20 | 6 | B |
| TAC126 | Shin-ike pond, Nagano | 2/8/89 | **41** | 25 | 31 | 32 | 24 | 31 | 26 | 28 | NA |
| TAC128 | Kamisawa-ike pond, Nagano | 2/8/89 | **42** | 26 | 32 | 33 | 25 | 32 | 27 | 29 | B |
| TAC129 | Kamisawa-ike pond, Nagano | 2/8/89 | **43** | 5 | 5 | 34 | 26 | 5 | 28 | 30 | A |
| TAC173 | Lake Shirakaba | 23/8/89 | **39** | 7 | 8 | 30 | 7 | 29 | 17 | 26 | D |
| TAC174 | Lake Shirakaba | 23/8/89 | **39** | 7 | 8 | 30 | 7 | 29 | 17 | 26 | D |
| TAC175 | Lake Shirakaba | 23/8/89 | **29** | 18 | 21 | 3 | 17 | 3 | 14 | 21 | A |
| TAC176 | Lake Shirakaba | 23/8/89 | **29** | 18 | 21 | 3 | 17 | 3 | 14 | 21 | A |
| TAC177 | Lake Shirakaba | 23/8/89 | **49** | 30 | 15 | 23 | 31 | 37 | 34 | 34 | A |
| TAC178 | Lake Shirakaba | 23/8/89 | **49** | 30 | 15 | 23 | 31 | 37 | 34 | 34 | A |
| TAC183 | Lake Shirakaba | 23/8/89 | **29** | 18 | 21 | 3 | 17 | 3 | 14 | 21 | A |
| TAC136 | Showa-Tameike pond, Fukuoka | 28/8/89 | **44** | 27 | 33 | 26 | 27 | 33 | 29 | 6 | B |
| TAC146 | Lake Ohnuma | 5/9/89 | **45** | 26 | 34 | 33 | 28 | 32 | 30 | 31 | B |
| TAC147 | Lake Ohnuma | 5/9/89 | **27** | 7 | 8 | 22 | 7 | 22 | 16 | 19 | D |
| TAC148 | Lake Ohnuma | 5/9/89 | **27** | 7 | 8 | 22 | 7 | 22 | 16 | 19 | D |
| TAC149 | Lake Ohnuma | 5/9/89 | **27** | 7 | 8 | 22 | 7 | 22 | 16 | 19 | D |
| TAC150 | Lake Ohnuma | 5/9/89 | **73** | 6 | 51 | 51 | 40 | 46 | 47 | 44 | B |
| TAC151 | Lake Ohnuma | 5/9/89 | **73** | 6 | 51 | 51 | 40 | 46 | 47 | 44 | B |
| TAC152 | Lake Ohnuma | 5/9/89 | **46** | 28 | 35 | 25 | 29 | 34 | 31 | 32 | F |
| TAC153 | Lake Ohnuma | 5/9/89 | **46** | 28 | 35 | 25 | 29 | 34 | 31 | 32 | F |
| TAC154 | Lake Tega | 13/9/89 | **75** | 38 | 53 | 53 | 42 | 48 | 49 | 46 | NA |
| TAC155 | Lake Tega | 13/9/89 | **76** | 39 | 54 | 54 | 43 | 49 | 14 | 47 | A |
| TAC156 | Lake Tega | 13/9/89 | **74** | 37 | 52 | 52 | 41 | 47 | 48 | 45 | NA |
| TAC157 | Lake Tega | 13/9/89 | **76** | 39 | 54 | 54 | 43 | 49 | 14 | 47 | A |
| TAC159 | Lake Tega | 13/9/89 | **48** | 20 | 37 | 36 | 8 | 36 | 33 | 24 | B |
| TAC160 | Lake Tega | 13/9/89 | **48** | 20 | 37 | 36 | 8 | 36 | 33 | 24 | B |
| TAC162 | Lake Tega | 13/9/89 | **26** | 9 | 15 | 21 | 12 | 16 | 8 | 8 | A |
| TAC163 | Lake Tega | 13/9/89 | **1** | 1 | 1 | 1 | 1 | 1 | 1 | 1 | E |
| TAC164 | Lake Tega | 13/9/89 | **1** | 1 | 1 | 1 | 1 | 1 | 1 | 1 | E |
| TAC182 | Lake Tega | 17/9/89 | **29** | 18 | 21 | 3 | 17 | 3 | 14 | 21 | A |
| TAC185 | Lake Tega | 17/9/89 | **48** | 20 | 37 | 36 | 8 | 36 | 33 | 24 | B |
| TAC165 | Lake Okutama | 17/9/89 | **55** | 31 | 41 | 3 | 3 | 3 | 35 | 37 | A |
| TAC166 | Lake Okutama | 17/9/89 | **4** | 4 | 4 | 4 | 4 | 4 | 4 | 4 | A |
| TAC167 | Lake Okutama | 17/9/89 | **55** | 31 | 41 | 3 | 3 | 3 | 35 | 37 | A |
| TAC169 | Lake Okutama | 17/9/89 | **56** | 32 | 42 | 39 | 33 | 40 | 36 | 38 | NA |
| TAC170 | Lake Okutama | 17/9/89 | **57** | 33 | 43 | 40 | 34 | 41 | 37 | 39 | "X" |
| TAC171 | Lake Okutama | 17/9/89 | **58** | 34 | 1 | 11 | 1 | 39 | 1 | 1 | E |
| TAC172 | Lake Okutama | 17/9/89 | **58** | 34 | 1 | 11 | 1 | 39 | 1 | 1 | E |
| TAC179 | Lake Okutama | 17/9/89 | **55** | 31 | 41 | 3 | 3 | 3 | 35 | 37 | A |
| TAC180 | Lake Okutama | 17/9/89 | **55** | 31 | 41 | 3 | 3 | 3 | 35 | 37 | A |
| TAC187 | Lake Okutama | 17/9/89 | **55** | 31 | 41 | 3 | 3 | 3 | 35 | 37 | A |
| TAC188 | Lake Okutama | 17/9/89 | **55** | 31 | 41 | 3 | 3 | 3 | 35 | 37 | A |
| TAC189 | Lake Okutama | 17/9/89 | **55** | 31 | 41 | 3 | 3 | 3 | 35 | 37 | A |
| TAC190 | Lake Okutama | 17/9/89 | **55** | 31 | 41 | 3 | 3 | 3 | 35 | 37 | A |
| TAC191 | Lake Okutama | 17/9/89 | **55** | 31 | 41 | 3 | 3 | 3 | 35 | 37 | A |
| TAC192 | Lake Okutama | 17/9/89 | **55** | 31 | 41 | 3 | 3 | 3 | 35 | 37 | A |
| TAC376 | Ishigaki Dam, Okinawa | 28/10/90 | **71** | 7 | 8 | 49 | 7 | 29 | 17 | 42 | D |
| TAC377 | Ishigaki Dam, Okinawa | 28/10/90 | **71** | 7 | 8 | 49 | 7 | 29 | 17 | 42 | D |
| TAC378 | Ishigaki Dam, Okinawa | 28/10/90 | **72** | 36 | 18 | 50 | 39 | 5 | 46 | 43 | A |
| TAC379 | Ishigaki Dam, Okinawa | 28/10/90 | **72** | 36 | 18 | 50 | 39 | 5 | 46 | 43 | A |
| TAC380 | Ishigaki Dam, Okinawa | 28/10/90 | **72** | 36 | 18 | 50 | 39 | 5 | 46 | 43 | A |
| TAC374 | Ishigaki Dam, Okinawa | 28/10/90 | **78** | 40 | 55 | 56 | 44 | 8 | 39 | 6 | B |
| TAC375 | Ishigaki Dam, Okinawa | 28/10/90 | **78** | 40 | 55 | 56 | 44 | 8 | 39 | 6 | B |
| TAC198 | Lake Barato | 27/8/90 | **50** | 12 | 14 | 37 | 32 | 38 | 13 | 34 | C |
| TAC199 | Lake Barato | 27/8/90 | **50** | 12 | 14 | 37 | 32 | 38 | 13 | 34 | C |
| TAC200 | Lake Barato | 27/8/90 | **50** | 12 | 14 | 37 | 32 | 38 | 13 | 34 | C |
| NIES1173 | Saburo-ike Pond, Kagawa | 17/9/90 | **4** | 4 | 4 | 4 | 4 | 4 | 4 | 4 | A |
| NIES1174 | Saburo-ike Pond, Kagawa | 17/9/90 | **188** | 30 | 15 | 23 | 87 | 94 | 89 | 34 | A |
| NIES1175 | Saburo-ike Pond, Kagawa | 17/9/90 | **189** | 10 | 106 | 111 | 9 | 95 | 9 | 77 | NA |
| NIES1176 | Saburo-ike Pond, Kagawa | 17/9/90 | **135** | 14 | 83 | 82 | 65 | 25 | 65 | 62 | NA |
| NIES1177 | Saburo-ike Pond, Kagawa | 17/9/90 | **190** | 14 | 23 | 112 | 88 | 43 | 90 | 44 | B |
| NIES1179 | Shin-ike pond, Kagawa | 17/9/90 | **191** | 11 | 11 | 10 | 1 | 14 | 1 | 1 | E |
| NIES1180 | Shin-ike pond, Kagawa | 17/9/90 | **26** | 9 | 15 | 21 | 12 | 16 | 8 | 8 | A |
| NIES1181 | Shin-ike pond, Kagawa | 17/9/90 | **192** | 6 | 51 | 51 | 40 | 46 | 91 | 6 | B |
| NIES1183 | Sakase-ike pond, Kagawa | 17/9/90 | **193** | 20 | 107 | 94 | 64 | 33 | 64 | 6 | B |
| NIES1184 | Sakase-ike pond, Kagawa | 17/9/90 | **193** | 20 | 107 | 94 | 64 | 33 | 64 | 6 | B |
| NIES1185 | Sakase-ike pond, Kagawa | 17/9/90 | **194** | 36 | 18 | 50 | 26 | 5 | 46 | 78 | A |
| NIES1186 | Sakase-ike pond, Kagawa | 17/9/90 | **194** | 36 | 18 | 50 | 26 | 5 | 46 | 78 | A |
| TAC395 | Lake Suwa | 31/3/90 | **17** | 12 | 14 | 14 | 11 | 15 | 13 | 12 | C |
| TAC396 | Lake Suwa | 31/3/90 | **53** | 7 | 39 | 7 | 7 | 7 | 7 | 7 | D |
| TAC401 | Lake Suwa | 31/3/90 | **8** | 7 | 8 | 7 | 7 | 7 | 7 | 7 | D |
| TAC402 | Lake Suwa | 31/3/90 | **8** | 7 | 8 | 7 | 7 | 7 | 7 | 7 | D |
| TAC381 | Tengan Dam, Okinawa | 27/10/90 | **68** | 6 | 49 | 47 | 20 | 45 | 43 | 6 | B |
| TAC382 | Tengan Dam, Okinawa | 27/10/90 | **33** | 6 | 25 | 26 | 20 | 8 | 17 | 6 | B |
| TAC383 | Tengan Dam, Okinawa | 27/10/90 | **79** | 7 | 8 | 57 | 7 | 22 | 17 | 26 | D |
| TAC384 | Tengan Dam, Okinawa | 27/10/90 | **79** | 7 | 8 | 57 | 7 | 22 | 17 | 26 | D |
| TAC385 | Tengan Dam, Okinawa | 27/10/90 | **72** | 36 | 18 | 50 | 39 | 5 | 46 | 43 | A |
| TAC386 | Tengan Dam, Okinawa | 27/10/90 | **72** | 36 | 18 | 50 | 39 | 5 | 46 | 43 | A |
| TAC371 | Fukuchi Dam, Okinawa | 27/10/90 | **72** | 36 | 18 | 50 | 39 | 5 | 46 | 43 | A |
| TAC372 | Fukuchi Dam, Okinawa | 27/10/90 | **72** | 36 | 18 | 50 | 39 | 5 | 46 | 43 | A |
| TAC373 | Fukuchi Dam, Okinawa | 27/10/90 | **72** | 36 | 18 | 50 | 39 | 5 | 46 | 43 | A |
| TAC387 | Maesato Dam, Okinawa | 28/10/90 | **79** | 7 | 8 | 57 | 7 | 22 | 17 | 26 | D |
| TAC388 | Maesato Dam, Okinawa | 28/10/90 | **79** | 7 | 8 | 57 | 7 | 22 | 17 | 26 | D |
| TAC389 | Maesato Dam, Okinawa | 28/10/90 | **79** | 7 | 8 | 57 | 7 | 22 | 17 | 26 | D |
| TAC355 | Kunma Dam, Okinawa | 26/10/90 | **68** | 6 | 49 | 47 | 20 | 45 | 43 | 6 | B |
| TAC356 | Kunma Dam, Okinawa | 26/10/90 | **69** | 20 | 50 | 26 | 20 | 8 | 44 | 6 | B |
| TAC357 | Kunma Dam, Okinawa | 26/10/90 | **70** | 22 | 32 | 48 | 20 | 8 | 45 | 6 | B |
| TAC358 | Kunma Dam, Okinawa | 26/10/90 | **71** | 7 | 8 | 49 | 7 | 29 | 17 | 42 | D |
| TAC359 | Kunma Dam, Okinawa | 26/10/90 | **71** | 7 | 8 | 49 | 7 | 29 | 17 | 42 | D |
| TAC360 | Kunma Dam, Okinawa | 26/10/90 | **71** | 7 | 8 | 49 | 7 | 29 | 17 | 42 | D |
| TAC361 | Kunma Dam, Okinawa | 26/10/90 | **72** | 36 | 18 | 50 | 39 | 5 | 46 | 43 | A |
| TAC362 | Kunma Dam, Okinawa | 26/10/90 | **72** | 36 | 18 | 50 | 39 | 5 | 46 | 43 | A |
| TAC363 | Kunma Dam, Okinawa | 26/10/90 | **72** | 36 | 18 | 50 | 39 | 5 | 46 | 43 | A |
| TAC364 | Tatsugami Dam, Okinawa | 27/10/90 | **33** | 6 | 25 | 26 | 20 | 8 | 17 | 6 | B |
| TAC365 | Tatsugami Dam, Okinawa | 27/10/90 | **33** | 6 | 25 | 26 | 20 | 8 | 17 | 6 | B |
| TAC368 | Tatsugami Dam, Okinawa | 27/10/90 | **71** | 7 | 8 | 49 | 7 | 29 | 17 | 42 | D |
| TAC369 | Tatsugami Dam, Okinawa | 27/10/90 | **72** | 36 | 18 | 50 | 39 | 5 | 46 | 43 | A |
| TAC370 | Tatsugami Dam, Okinawa | 27/10/90 | **72** | 36 | 18 | 50 | 39 | 5 | 46 | 43 | A |
| TAC350 | Lake Toro | 28/9/90 | **32** | 5 | 24 | 5 | 10 | 5 | 21 | 5 | A |
| TAC352 | Lake Tofutsu | 28/9/90 | **47** | 29 | 36 | 35 | 30 | 35 | 32 | 33 | NA |
| NIES903 | Hubei, China | */7/96 | **26** | 9 | 15 | 21 | 12 | 16 | 8 | 8 | A |
| CL4 | Hubei, China | */*/96 | **19** | 9 | 15 | 15 | 12 | 16 | 14 | 8 | A |
| NIES904 | Bangkok, Thailand | */10/96 | **54** | 11 | 40 | 13 | 1 | 39 | 1 | 1 | E |
| T20-3 | Nakhon Pathom, Thailand | */*/96 | **23** | 16 | 19 | 19 | 15 | 20 | 15 | 17 | "X" |
| NIES901 | Dundee, Scotland | */8/97 | **59** | 7 | 8 | 41 | 7 | 29 | 38 | 18 | D |
| NIES843 | Lake Kasumigaura | 28/8/97 | **18** | 9 | 15 | 15 | 12 | 16 | 14 | 13 | A |
| TL2 | Chon Bri, Thailand | */1/97 | **22** | 15 | 18 | 18 | 5 | 19 | 11 | 16 | A |
| MCS3 | Phuket, Thailand | */*/00 | **20** | 13 | 16 | 16 | 13 | 17 | 3 | 14 | A |
| KI-1 | Tsuchiura, Lake Kasumigaura | 22/8/04 | **103** | 20 | 62 | 71 | 36 | 33 | 58 | 6 | B |
| Kw1 | Tsuchiura, Lake Kasumigaura | 22/8/04 | **16** | 11 | 12 | 13 | 1 | 14 | 1 | 1 | E |
| Kw3 | Tsuchiura, Lake Kasumigaura | 22/8/04 | **1** | 1 | 1 | 1 | 1 | 1 | 1 | 1 | E |
| Kw4 | Tsuchiura, Lake Kasumigaura | 22/8/04 | **105** | 1 | 1 | 11 | 1 | 1 | 1 | 1 | E |
| Kw5 | Tsuchiura, Lake Kasumigaura | 22/8/04 | **105** | 1 | 1 | 11 | 1 | 1 | 1 | 1 | E |
| Kw7 | Tsuchiura, Lake Kasumigaura | 1/9/04 | **1** | 1 | 1 | 1 | 1 | 1 | 1 | 1 | E |
| Kw8 | Tsuchiura, Lake Kasumigaura | 1/9/04 | **1** | 1 | 1 | 1 | 1 | 1 | 1 | 1 | E |
| KA3b | Tsuchiura, Lake Kasumigaura | 1/9/04 | **80** | 39 | 56 | 58 | 45 | 51 | 50 | 48 | A |
| KA4 | Tsuchiura, Lake Kasumigaura | 1/9/04 | **81** | 41 | 18 | 59 | 5 | 5 | 16 | 49 | A |
| KA5c | Tsuchiura, Lake Kasumigaura | 1/9/04 | **103** | 20 | 62 | 71 | 36 | 33 | 58 | 6 | B |
| KI-3 | Tsuchiura, Lake Kasumigaura | 1/9/04 | **104** | 2 | 63 | 72 | 2 | 60 | 59 | 2 | NA |
| KA6 | Futto, Lake Kasumigaura | 9/9/04 | **82** | 5 | 5 | 5 | 26 | 5 | 11 | 50 | A |
| KS1 | Futto, Lake Kasumigaura | 9/9/04 | **83** | 20 | 57 | 60 | 20 | 52 | 45 | 6 | B |
| SI-2 | Funato-bashi, Lake Suwa | 7/10/04 | **107** | 26 | 32 | 73 | 20 | 61 | 6 | 25 | B |
| Sw1 | Funato-bashi, Lake Suwa | 7/10/04 | **67** | 12 | 48 | 46 | 11 | 15 | 42 | 41 | C |
| Sw2 | Funato-bashi, Lake Suwa | 7/10/04 | **67** | 12 | 48 | 46 | 11 | 15 | 42 | 41 | C |
| Sw3 | Funato-bashi, Lake Suwa | 7/10/04 | **67** | 12 | 48 | 46 | 11 | 15 | 42 | 41 | C |
| Sw4 | Funato-bashi, Lake Suwa | 7/10/04 | **85** | 9 | 15 | 15 | 46 | 16 | 8 | 8 | A |
| Sw5 | Funato-bashi, Lake Suwa | 7/10/04 | **85** | 9 | 15 | 15 | 46 | 16 | 8 | 8 | A |
| Sw7 | Funato-bashi, Lake Suwa | 7/10/04 | **67** | 12 | 48 | 46 | 11 | 15 | 42 | 41 | C |
| Sw8 | Mizube Park, Lake Suwa | 7/10/04 | **67** | 12 | 48 | 46 | 11 | 15 | 42 | 41 | C |
| Sw9 | Mizube Park, Lake Suwa | 7/10/04 | **67** | 12 | 48 | 46 | 11 | 15 | 42 | 41 | C |
| Sw10 | Mizube Park, Lake Suwa | 7/10/04 | **67** | 12 | 48 | 46 | 11 | 15 | 42 | 41 | C |
| Sw11 | Mizube Park, Lake Suwa | 7/10/04 | **67** | 12 | 48 | 46 | 11 | 15 | 42 | 41 | C |
| Sw12 | Mizube Park, Lake Suwa | 7/10/04 | **67** | 12 | 48 | 46 | 11 | 15 | 42 | 41 | C |
| Sw13 | Mizube Park, Lake Suwa | 7/10/04 | **67** | 12 | 48 | 46 | 11 | 15 | 42 | 41 | C |
| Sw14 | Mizube Park, Lake Suwa | 7/10/04 | **67** | 12 | 48 | 46 | 11 | 15 | 42 | 41 | C |
| Sw15 | Mizube Park, Lake Suwa | 7/10/04 | **67** | 12 | 48 | 46 | 11 | 15 | 42 | 41 | C |
| Sw16 | Mizube Park, Lake Suwa | 7/10/04 | **67** | 12 | 48 | 46 | 11 | 15 | 42 | 41 | C |
| Sw18 | Mizube Park, Lake Suwa | 7/10/04 | **67** | 12 | 48 | 46 | 11 | 15 | 42 | 41 | C |
| SA1 | Mizube Park, Lake Suwa | 7/10/04 | **106** | 39 | 58 | 61 | 43 | 53 | 11 | 51 | A |
| SA2 | Mizube Park, Lake Suwa | 7/10/04 | **84** | 39 | 58 | 61 | 43 | 53 | 51 | 51 | A |
| SHA4 | Lake Shirakaba | 8/10/04 | **108** | 43 | 4 | 66 | 12 | 4 | 18 | 58 | A |
| Ks05TA1 | Takasaki, Lake Kasumigaura | 17/7/05 | **109** | 19 | 64 | 74 | 51 | 62 | 60 | 59 | G |
| Ks05TA2 | Takasaki, Lake Kasumigaura | 17/7/05 | **110** | 19 | 65 | 75 | 52 | 62 | 61 | 60 | G |
| Ks05TA3 | Takasaki, Lake Kasumigaura | 17/7/05 | **111** | 45 | 66 | 75 | 53 | 63 | 61 | 59 | G |
| Ks05TA4 | Takasaki, Lake Kasumigaura | 17/7/05 | **112** | 19 | 64 | 76 | 51 | 62 | 22 | 59 | G |
| Ks05TA5 | Takasaki, Lake Kasumigaura | 17/7/05 | **113** | 45 | 67 | 75 | 54 | 63 | 60 | 59 | G |
| Ks05TA6 | Takasaki, Lake Kasumigaura | 17/7/05 | **114** | 19 | 68 | 75 | 55 | 63 | 62 | 59 | G |
| Ks05TA10 | Takasaki, Lake Kasumigaura | 17/7/05 | **112** | 19 | 64 | 76 | 51 | 62 | 22 | 59 | G |
| Ks05TA11 | Takasaki, Lake Kasumigaura | 17/7/05 | **115** | 45 | 69 | 75 | 52 | 64 | 62 | 59 | G |
| Ks05TA21 | Takasaki, Lake Kasumigaura | 17/7/05 | **116** | 45 | 69 | 75 | 52 | 65 | 62 | 59 | G |
| Ks05TA22 | Takasaki, Lake Kasumigaura | 17/7/05 | **117** | 45 | 66 | 75 | 52 | 63 | 62 | 59 | G |
| Ks05TA23 | Takasaki, Lake Kasumigaura | 17/7/05 | **118** | 19 | 70 | 77 | 55 | 66 | 62 | 59 | G |
| Ks05TA25 | Takasaki, Lake Kasumigaura | 17/7/05 | **119** | 45 | 71 | 75 | 53 | 63 | 61 | 59 | G |
| Ks05TA26 | Takasaki, Lake Kasumigaura | 17/7/05 | **117** | 45 | 66 | 75 | 52 | 63 | 62 | 59 | G |
| Ks05TA27 | Takasaki, Lake Kasumigaura | 17/7/05 | **120** | 19 | 65 | 77 | 52 | 62 | 61 | 60 | G |
| Ks05TA28 | Takasaki, Lake Kasumigaura | 17/7/05 | **121** | 45 | 72 | 75 | 56 | 64 | 62 | 59 | G |
| Ks05TA30 | Takasaki, Lake Kasumigaura | 17/7/05 | **119** | 45 | 71 | 75 | 53 | 63 | 61 | 59 | G |
| Ks05TA31 | Takasaki, Lake Kasumigaura | 17/7/05 | **122** | 45 | 73 | 75 | 52 | 65 | 60 | 59 | G |
| Ks05TA32 | Takasaki, Lake Kasumigaura | 17/7/05 | **123** | 45 | 74 | 75 | 57 | 63 | 61 | 59 | G |
| Ks05TA33 | Takasaki, Lake Kasumigaura | 17/7/05 | **124** | 45 | 75 | 75 | 57 | 63 | 61 | 59 | G |
| Ks05TA34 | Takasaki, Lake Kasumigaura | 17/7/05 | **110** | 19 | 65 | 75 | 52 | 62 | 61 | 60 | G |
| Ks05TA35 | Takasaki, Lake Kasumigaura | 17/7/05 | **125** | 45 | 76 | 75 | 58 | 63 | 62 | 59 | G |
| Ks05TA36 | Takasaki, Lake Kasumigaura | 17/7/05 | **112** | 19 | 64 | 76 | 51 | 62 | 22 | 59 | G |
| Ks05TA37 | Takasaki, Lake Kasumigaura | 17/7/05 | **126** | 45 | 77 | 78 | 59 | 63 | 60 | 59 | G |
| Ks05TA38 | Takasaki, Lake Kasumigaura | 17/7/05 | **120** | 19 | 65 | 77 | 52 | 62 | 61 | 60 | G |
| Ks05TA41 | Takasaki, Lake Kasumigaura | 17/7/05 | **124** | 45 | 75 | 75 | 57 | 63 | 61 | 59 | G |
| Ks05TA43 | Takasaki, Lake Kasumigaura | 17/7/05 | **127** | 45 | 78 | 75 | 52 | 63 | 62 | 59 | G |
| Ks05TA44 | Takasaki, Lake Kasumigaura | 17/7/05 | **119** | 45 | 71 | 75 | 53 | 63 | 61 | 59 | G |
| Ks05TA46 | Takasaki, Lake Kasumigaura | 17/7/05 | **128** | 45 | 79 | 75 | 57 | 63 | 61 | 59 | G |
| Ks05TA49 | Takasaki, Lake Kasumigaura | 17/7/05 | **110** | 19 | 65 | 75 | 52 | 62 | 61 | 60 | G |
| Ks05TA51 | Takasaki, Lake Kasumigaura | 17/7/05 | **86** | 35 | 59 | 62 | 47 | 54 | 52 | 25 | B |
| Ks05TA52 | Takasaki, Lake Kasumigaura | 17/7/05 | **129** | 19 | 80 | 79 | 60 | 63 | 60 | 59 | G |
| Ks05TA53 | Takasaki, Lake Kasumigaura | 17/7/05 | **117** | 45 | 66 | 75 | 52 | 63 | 62 | 59 | G |
| Ks05TA56 | Takasaki, Lake Kasumigaura | 17/7/05 | **130** | 46 | 81 | 80 | 61 | 67 | 16 | 61 | NA |
| Ks05TA57 | Takasaki, Lake Kasumigaura | 17/7/05 | **115** | 45 | 69 | 75 | 52 | 64 | 62 | 59 | G |
| Kv05TA01 | Takasaki, Lake Kasumigaura | 17/7/05 | **119** | 45 | 71 | 75 | 53 | 63 | 61 | 59 | G |
| Ks05TA61 | Takasaki, Lake Kasumigaura | 3/9/05 | **26** | 9 | 15 | 21 | 12 | 16 | 8 | 8 | A |
| Ks05TA62 | Takasaki, Lake Kasumigaura | 3/9/05 | **87** | 39 | 47 | 54 | 43 | 49 | 34 | 48 | A |
| Kw05TA03 | Takasaki, Lake Kasumigaura | 3/9/05 | **131** | 10 | 82 | 9 | 62 | 68 | 9 | 9 | NA |
| Kw05TA04 | Takasaki, Lake Kasumigaura | 3/9/05 | **132** | 22 | 11 | 26 | 1 | 33 | 39 | 21 | B |
| Ki05TA02 | Takasaki, Lake Kasumigaura | 3/9/05 | **88** | 14 | 27 | 63 | 48 | 55 | 53 | 25 | B |
| Ki05TA03 | Takasaki, Lake Kasumigaura | 3/9/05 | **133** | 20 | 33 | 26 | 63 | 33 | 63 | 6 | B |
| Ki05TA05 | Takasaki, Lake Kasumigaura | 3/9/05 | **134** | 20 | 33 | 81 | 64 | 33 | 64 | 6 | B |
| Ki05TA07 | Takasaki, Lake Kasumigaura | 3/9/05 | **89** | 6 | 60 | 64 | 48 | 55 | 53 | 25 | B |
| Ki05TA10 | Takasaki, Lake Kasumigaura | 3/9/05 | **135** | 14 | 83 | 82 | 65 | 25 | 65 | 62 | NA |
| Ki05TA11 | Takasaki, Lake Kasumigaura | 3/9/05 | **136** | 20 | 33 | 83 | 63 | 36 | 66 | 6 | B |
| Ks05IS01 | Iseki, Lake Kasumigaura | 17/7/05 | **137** | 20 | 84 | 60 | 20 | 52 | 45 | 6 | B |
| Ks05IS02 | Iseki, Lake Kasumigaura | 17/7/05 | **90** | 4 | 61 | 4 | 43 | 4 | 8 | 8 | A |
| Ks05IS04 | Iseki, Lake Kasumigaura | 17/7/05 | **138** | 11 | 11 | 10 | 1 | 69 | 1 | 1 | E |
| Ks05IS05 | Iseki, Lake Kasumigaura | 17/7/05 | **139** | 22 | 85 | 84 | 19 | 33 | 67 | 21 | B |
| Ks05IS06 | Iseki, Lake Kasumigaura | 17/7/05 | **140** | 47 | 86 | 85 | 66 | 70 | 68 | 63 | NA |
| Ks05IS11 | Iseki, Lake Kasumigaura | 17/7/05 | **91** | 35 | 59 | 62 | 49 | 54 | 54 | 6 | B |
| Ks05IS12 | Iseki, Lake Kasumigaura | 17/7/05 | **141** | 35 | 59 | 86 | 65 | 54 | 54 | 25 | B |
| Ks05IS14 | Iseki, Lake Kasumigaura | 17/7/05 | **142** | 20 | 59 | 62 | 47 | 54 | 52 | 25 | B |
| Ks05IS16 | Iseki, Lake Kasumigaura | 17/7/05 | **86** | 35 | 59 | 62 | 47 | 54 | 52 | 25 | B |
| Ks05IS17 | Iseki, Lake Kasumigaura | 17/7/05 | **143** | 26 | 32 | 33 | 25 | 32 | 6 | 6 | B |
| Ks05IS19 | Iseki, Lake Kasumigaura | 17/7/05 | **102** | 35 | 59 | 62 | 47 | 54 | 23 | 25 | B |
| Kn05IS01 | Iseki, Lake Kasumigaura | 17/7/05 | **87** | 39 | 47 | 54 | 43 | 49 | 34 | 48 | A |
| Ks05YA03 | Yasuzuka, Lake Kitaura | 14/8/05 | **144** | 7 | 8 | 49 | 7 | 7 | 69 | 18 | D |
| Ks05YA05 | Yasuzuka, Lake Kitaura | 14/8/05 | **85** | 9 | 15 | 15 | 46 | 16 | 8 | 8 | A |
| Ks05YA11 | Yasuzuka, Lake Kitaura | 14/8/05 | **92** | 39 | 56 | 58 | 50 | 51 | 55 | 48 | A |
| Ki05YA01 | Yasuzuka, Lake Kitaura | 14/8/05 | **145** | 48 | 36 | 87 | 67 | 71 | 32 | 64 | NA |
| Ki05YA02 | Yasuzuka, Lake Kitaura | 14/8/05 | **146** | 49 | 87 | 88 | 68 | 72 | 70 | 65 | NA |
| Ki05YA03 | Yasuzuka, Lake Kitaura | 14/8/05 | **147** | 50 | 63 | 2 | 2 | 73 | 59 | 2 | NA |
| Ki05YA04 | Yasuzuka, Lake Kitaura | 14/8/05 | **148** | 20 | 88 | 89 | 6 | 74 | 71 | 6 | B |
| Kv05YA06 | Yasuzuka, Lake Kitaura | 14/8/05 | **26** | 9 | 15 | 21 | 12 | 16 | 8 | 8 | A |
| Kw05YA03 | Yasuzuka, Lake Kitaura | 14/8/05 | **149** | 51 | 89 | 90 | 69 | 75 | 72 | 66 | NA |
| Kw05YA04 | Yasuzuka, Lake Kitaura | 14/8/05 | **150** | 1 | 90 | 91 | 1 | 39 | 1 | 1 | E |
| Tw05AK01 | Akebono-bashi, Lake Teganuma | 10/9/05 | **151** | 52 | 91 | 25 | 29 | 76 | 73 | 67 | F |
| Tw05AK02 | Akebono-bashi, Lake Teganuma | 10/9/05 | **152** | 11 | 92 | 92 | 43 | 14 | 1 | 1 | E |
| Tw05AK03 | Akebono-bashi, Lake Teganuma | 10/9/05 | **151** | 52 | 91 | 25 | 29 | 76 | 73 | 67 | F |
| Tw05AK04 | Akebono-bashi, Lake Teganuma | 10/9/05 | **151** | 52 | 91 | 25 | 29 | 76 | 73 | 67 | F |
| Tw05AK06 | Akebono-bashi, Lake Teganuma | 10/9/05 | **152** | 11 | 92 | 92 | 43 | 14 | 1 | 1 | E |
| Tw05AK10 | Akebono-bashi, Lake Teganuma | 10/9/05 | **153** | 53 | 93 | 93 | 29 | 77 | 74 | 67 | F |
| Tw05AK11 | Akebono-bashi, Lake Teganuma | 10/9/05 | **154** | 53 | 94 | 25 | 29 | 78 | 31 | 67 | F |
| Tn05AK01 | Akebono-bashi, Lake Teganuma | 10/9/05 | **93** | 42 | 16 | 65 | 13 | 56 | 56 | 52 | A |
| Tn05AK02 | Akebono-bashi, Lake Teganuma | 10/9/05 | **94** | 43 | 4 | 66 | 43 | 4 | 8 | 4 | A |
| Tn05AK03 | Akebono-bashi, Lake Teganuma | 10/9/05 | **95** | 33 | 43 | 67 | 20 | 41 | 57 | 53 | "X" |
| Tn05AK05 | Akebono-bashi, Lake Teganuma | 10/9/05 | **96** | 39 | 56 | 68 | 43 | 57 | 55 | 48 | A |
| Ii05FU01 | Futago-Bashi, Lake Inba | 19/9/05 | **155** | 20 | 49 | 94 | 64 | 33 | 75 | 6 | B |
| Iw05FU01 | Futago-Bashi, Lake Inba | 19/9/05 | **156** | 1 | 1 | 11 | 70 | 14 | 1 | 1 | E |
| Iw05FU06 | Futago-Bashi, Lake Inba | 19/9/05 | **157** | 11 | 90 | 10 | 1 | 14 | 1 | 1 | E |
| Iw05FU08 | Futago-Bashi, Lake Inba | 19/9/05 | **157** | 11 | 90 | 10 | 1 | 14 | 1 | 1 | E |
| In05FU04 | Futago-Bashi, Lake Inba | 19/9/05 | **97** | 44 | 15 | 69 | 50 | 58 | 8 | 54 | A |
| Ia05Yo03 | Yoshitaka, Lake Inba | 19/9/05 | **98** | 30 | 15 | 5 | 50 | 59 | 38 | 55 | A |
| Ia05Yo05 | Yoshitaka, Lake Inba | 19/9/05 | **99** | 30 | 15 | 23 | 50 | 59 | 38 | 55 | A |
| Ia05Yo06 | Yoshitaka, Lake Inba | 19/9/05 | **158** | 54 | 36 | 95 | 71 | 79 | 32 | 3 | NA |
| Ii05Yo01 | Yoshitaka, Lake Inba | 19/9/05 | **159** | 20 | 95 | 96 | 72 | 74 | 76 | 24 | B |
| Ii05Yo02 | Yoshitaka, Lake Inba | 19/9/05 | **160** | 6 | 7 | 55 | 6 | 50 | 71 | 6 | B |
| Ii05Yo03 | Yoshitaka, Lake Inba | 19/9/05 | **155** | 20 | 49 | 94 | 64 | 33 | 75 | 6 | B |
| Ii05Yo04 | Yoshitaka, Lake Inba | 19/9/05 | **148** | 20 | 88 | 89 | 6 | 74 | 71 | 6 | B |
| In05Yo02 | Yoshitaka, Lake Inba | 19/9/05 | **97** | 44 | 15 | 69 | 50 | 58 | 8 | 54 | A |
| In05Yo05 | Yoshitaka, Lake Inba | 19/9/05 | **97** | 44 | 15 | 69 | 50 | 58 | 8 | 54 | A |
| In05Yo06 | Yoshitaka, Lake Inba | 19/9/05 | **97** | 44 | 15 | 69 | 50 | 58 | 8 | 54 | A |
| In05Yo08 | Yoshitaka, Lake Inba | 19/9/05 | **97** | 44 | 15 | 69 | 50 | 58 | 8 | 54 | A |
| In05Yo09 | Yoshitaka, Lake Inba | 19/9/05 | **97** | 44 | 15 | 69 | 50 | 58 | 8 | 54 | A |
| Iw05Yo08 | Yoshitaka, Lake Inba | 19/9/05 | **161** | 28 | 91 | 97 | 73 | 80 | 74 | 68 | F |
| Sw05Mb09 | Mizube Park, Lake Suwa | 28/9/05 | **67** | 12 | 48 | 46 | 11 | 15 | 42 | 41 | C |
| Sw05Mb10 | Mizube Park, Lake Suwa | 28/9/05 | **67** | 12 | 48 | 46 | 11 | 15 | 42 | 41 | C |
| Sw05Mb11 | Mizube Park, Lake Suwa | 28/9/05 | **67** | 12 | 48 | 46 | 11 | 15 | 42 | 41 | C |
| Sn05Mb03 | Mizube Park, Lake Suwa | 28/9/05 | **100** | 36 | 18 | 70 | 26 | 5 | 46 | 56 | A |
| Sn05Mb05 | Mizube Park, Lake Suwa | 28/9/05 | **100** | 36 | 18 | 70 | 26 | 5 | 46 | 56 | A |
| Sn05Mb06 | Mizube Park, Lake Suwa | 28/9/05 | **163** | 36 | 18 | 50 | 10 | 5 | 77 | 30 | A |
| Ss05Mb06 | Mizube Park, Lake Suwa | 28/9/05 | **164** | 49 | 96 | 99 | 68 | 81 | 78 | 65 | NA |
| Sw05Hb04 | Yacht harbor, Lake Suwa | 29/9/05 | **67** | 12 | 48 | 46 | 11 | 15 | 42 | 41 | C |
| Sw05Hb08 | Yacht harbor, Lake Suwa | 29/9/05 | **67** | 12 | 48 | 46 | 11 | 15 | 42 | 41 | C |
| Sn05Hb06 | Yacht harbor, Lake Suwa | 29/9/05 | **162** | 7 | 8 | 98 | 7 | 22 | 17 | 18 | D |
| Thvi7 | Chiang Mai, Thailand | 25/5/06 | **165** | 55 | 97 | 100 | 74 | 82 | 79 | 69 | NA |
| Thvi8 | Chiang Mai, Thailand | 25/5/06 | **166** | 56 | 98 | 101 | 2 | 60 | 80 | 70 | NA |
| LNN-s1 | Nam Ngum Dam, Laos | 23/12/06 | **101** | 15 | 18 | 18 | 5 | 5 | 11 | 57 | A |
| LNN-s5 | Nam Ngum Dam, Laos | 23/12/06 | **101** | 15 | 18 | 18 | 5 | 5 | 11 | 57 | A |
| LNN-s6 | Nam Ngum Dam, Laos | 23/12/06 | **101** | 15 | 18 | 18 | 5 | 5 | 11 | 57 | A |
| CTS3-5 | Lake Tonlé Sap, Cambodia | 21/2/07 | **167** | 57 | 99 | 102 | 75 | 83 | 81 | 71 | NA |
| CTS3-8 | Lake Tonlé Sap, Cambodia | 21/2/07 | **168** | 58 | 100 | 103 | 76 | 84 | 82 | 72 | NA |
| Ii07Yo01 | Yoshitaka, Lake Inba | 4/7/07 | **195** | 20 | 108 | 113 | 6 | 96 | 29 | 6 | B |
| Ii07Yo02 | Yoshitaka, Lake Inba | 4/7/07 | **196** | 14 | 109 | 64 | 48 | 55 | 53 | 25 | B |
| Is07Yo01 | Yoshitaka, Lake Inba | 4/7/07 | **197** | 63 | 110 | 6 | 6 | 97 | 29 | 6 | B |
| Ks07TS11 | Tsuchiura, Lake Kasumigaura | 3/8/07 | **122** | 45 | 73 | 75 | 52 | 65 | 60 | 59 | G |
| Ks07TS13 | Tsuchiura, Lake Kasumigaura | 3/8/07 | **127** | 45 | 78 | 75 | 52 | 63 | 62 | 59 | G |
| Ks07TS20 | Tsuchiura, Lake Kasumigaura | 3/8/07 | **169** | 19 | 101 | 77 | 77 | 85 | 62 | 59 | G |
| Ks07TS27 | Tsuchiura, Lake Kasumigaura | 3/8/07 | **170** | 19 | 70 | 77 | 55 | 86 | 62 | 59 | G |
| Ks07TS29 | Tsuchiura, Lake Kasumigaura | 3/8/07 | **171** | 19 | 65 | 77 | 52 | 62 | 62 | 60 | G |
| Ks07TS43 | Tsuchiura, Lake Kasumigaura | 9/8/07 | **172** | 45 | 64 | 76 | 52 | 65 | 62 | 59 | G |
| Ks07TS44 | Tsuchiura, Lake Kasumigaura | 9/8/07 | **172** | 45 | 64 | 76 | 52 | 65 | 62 | 59 | G |
| Ks07TS48 | Tsuchiura, Lake Kasumigaura | 9/8/07 | **173** | 45 | 102 | 74 | 52 | 66 | 60 | 59 | G |
| Ks07TS52c | Tsuchiura, Lake Kasumigaura | 9/8/07 | **174** | 59 | 96 | 104 | 68 | 72 | 78 | 65 | NA |
| Kn07TS91 | Tsuchiura, Lake Kasumigaura | 3/9/07 | **185** | 30 | 15 | 23 | 84 | 91 | 38 | 34 | A |
| Ks07TS93 | Tsuchiura, Lake Kasumigaura | 3/9/07 | **175** | 19 | 79 | 77 | 78 | 86 | 83 | 59 | G |
| Ks07TS99 | Tsuchiura, Lake Kasumigaura | 3/9/07 | **176** | 19 | 68 | 105 | 79 | 66 | 62 | 59 | G |
| Kw07TS101 | Tsuchiura, Lake Kasumigaura | 9/9/07 | **187** | 47 | 86 | 85 | 86 | 93 | 68 | 76 | NA |
| Ks07TS102 | Tsuchiura, Lake Kasumigaura | 9/9/07 | **177** | 19 | 102 | 74 | 80 | 66 | 60 | 59 | G |
| Ks07TS105 | Tsuchiura, Lake Kasumigaura | 9/9/07 | **178** | 19 | 66 | 75 | 52 | 65 | 62 | 59 | G |
| Ks07TS111 | Tsuchiura, Lake Kasumigaura | 9/9/07 | **117** | 45 | 66 | 75 | 52 | 63 | 62 | 59 | G |
| Kn07TS121 | Tsuchiura, Lake Kasumigaura | 16/9/07 | **186** | 62 | 105 | 110 | 85 | 92 | 88 | 75 | NA |
| Ks07TS123 | Tsuchiura, Lake Kasumigaura | 16/9/07 | **179** | 45 | 102 | 75 | 52 | 65 | 62 | 59 | G |
| Ks07TS127 | Tsuchiura, Lake Kasumigaura | 16/9/07 | **180** | 45 | 76 | 106 | 52 | 64 | 62 | 59 | G |
| Ks07TS131 | Tsuchiura, Lake Kasumigaura | 22/9/07 | **81** | 41 | 18 | 59 | 5 | 5 | 16 | 49 | A |
| Ks07TS132 | Tsuchiura, Lake Kasumigaura | 22/9/07 | **81** | 41 | 18 | 59 | 5 | 5 | 16 | 49 | A |
| Ks07TS134 | Tsuchiura, Lake Kasumigaura | 22/9/07 | **81** | 41 | 18 | 59 | 5 | 5 | 16 | 49 | A |
| Ks07TS137 | Tsuchiura, Lake Kasumigaura | 22/9/07 | **181** | 46 | 81 | 80 | 61 | 87 | 84 | 61 | NA |
| Ks07TS139 | Tsuchiura, Lake Kasumigaura | 22/9/07 | **182** | 19 | 103 | 107 | 81 | 88 | 85 | 28 | G |
| Ks07TS141 | Tsuchiura, Lake Kasumigaura | 7/10/07 | **183** | 60 | 104 | 108 | 82 | 89 | 86 | 73 | NA |
| Ks07TS143 | Tsuchiura, Lake Kasumigaura | 7/10/07 | **177** | 19 | 102 | 74 | 80 | 66 | 60 | 59 | G |
| Ks07TS156 | Tsuchiura, Lake Kasumigaura | 14/10/07 | **179** | 45 | 102 | 75 | 52 | 65 | 62 | 59 | G |
| Ks07TS157 | Tsuchiura, Lake Kasumigaura | 14/10/07 | **177** | 19 | 102 | 74 | 80 | 66 | 60 | 59 | G |
| Ks07TS159 | Tsuchiura, Lake Kasumigaura | 14/10/07 | **184** | 61 | 81 | 109 | 83 | 90 | 87 | 74 | NA |
| Sn07Hb02 | Yacht harbor, Lake Suwa | 25/9/07 | **198** | 39 | 111 | 114 | 89 | 98 | 34 | 79 | A |
| Sw07Hb06 | Yacht harbor, Lake Suwa | 25/9/07 | **199** | 12 | 112 | 115 | 11 | 99 | 13 | 34 | C |
| Sw07Hb16 | Yacht harbor, Lake Suwa | 25/9/07 | **199** | 12 | 112 | 115 | 11 | 99 | 13 | 34 | C |
| Sw07Km13 | Kamaguchi Floodgate , Lake Suwa | 26/9/07 | **199** | 12 | 112 | 115 | 11 | 99 | 13 | 34 | C |
| Sw07Km21 | Kamaguchi Floodgate , Lake Suwa | 26/9/07 | **199** | 12 | 112 | 115 | 11 | 99 | 13 | 34 | C |
| Hs07SP05 | Hamamatsu, Shizuoka | 22/10/07 | **200** | 48 | 36 | 116 | 67 | 100 | 32 | 64 | NA |
| Hw07SP01 | Hamamatsu, Shizuoka | 22/10/07 | **201** | 12 | 14 | 116 | 32 | 99 | 13 | 80 | C |
| Hw07SP02 | Hamamatsu, Shizuoka | 22/10/07 | **201** | 12 | 14 | 116 | 32 | 99 | 13 | 80 | C |
| Bi07BH01 | Ootsu, Lake Biwa | 12/11/07 | **103** | 20 | 62 | 71 | 36 | 33 | 58 | 6 | B |
| Bs07BH02 | Ootsu, Lake Biwa | 12/11/07 | **202** | 30 | 15 | 23 | 50 | 59 | 38 | 81 | A |
| Bv07BH03 | Ootsu, Lake Biwa | 12/11/07 | **203** | 4 | 4 | 4 | 4 | 4 | 92 | 4 | A |
| Ys07BZ04 | Lake Yogo | 13/11/07 | **204** | 36 | 5 | 117 | 26 | 5 | 46 | 30 | A |
| Rs08SH03 | Sokobaru Dam, Okinawa | 19/2/08 | **205** | 33 | 43 | 67 | 34 | 41 | 57 | 39 | "X" |
| Rs08SH05 | Sokobaru Dam, Okinawa | 19/2/08 | **206** | 33 | 113 | 118 | 90 | 101 | 93 | 82 | "X" |
| Rs08SH06 | Sokobaru Dam, Okinawa | 19/2/08 | **205** | 33 | 43 | 67 | 34 | 41 | 57 | 39 | "X" |
| Rs08NA05 | Nagura Dam, Okinawa | 20/2/08 | **206** | 33 | 113 | 118 | 90 | 101 | 93 | 82 | "X" |
| Rw08NA01 | Nagura Dam, Okinawa | 20/2/08 | **207** | 24 | 30 | 119 | 23 | 102 | 94 | 83 | NA |
| SKs08Zn11 | Jin-ike, Kagawa | 22/6/08 | **208** | 46 | 81 | 120 | 61 | 90 | 84 | 84 | NA |
| SKw08Ya02 | Yamashita-ike, Kagawa | 2/7/08 | **199** | 12 | 112 | 115 | 11 | 99 | 13 | 34 | C |
| SKw08Ya04 | Yamashita-ike, Kagawa | 2/7/08 | **209** | 10 | 114 | 111 | 91 | 68 | 9 | 9 | NA |
| Ki08TS01 | Tsuchiura, Lake Kasumigaura | 2/8/08 | **210** | 64 | 115 | 121 | 2 | 103 | 95 | 85 | NA |
| Ki08TS02 | Tsuchiura, Lake Kasumigaura | 2/8/08 | **211** | 65 | 116 | 122 | 92 | 104 | 67 | 86 | NA |
| Ka08TS01 | Tsuchiura, Lake Kasumigaura | 2/8/08 | **212** | 19 | 117 | 123 | 52 | 66 | 62 | 59 | G |
| Ka08TS02 | Tsuchiura, Lake Kasumigaura | 2/8/08 | **213** | 19 | 101 | 124 | 55 | 85 | 22 | 59 | G |
| Ka08TS03 | Tsuchiura, Lake Kasumigaura | 2/8/08 | **214** | 43 | 4 | 21 | 93 | 4 | 18 | 8 | A |
| Ks08TS03 | Tsuchiura, Lake Kasumigaura | 2/8/08 | **215** | 45 | 69 | 125 | 52 | 65 | 62 | 59 | G |
| Ks08TS05 | Tsuchiura, Lake Kasumigaura | 2/8/08 | **216** | 60 | 118 | 126 | 82 | 89 | 62 | 87 | NA |
| Ks08TS06 | Tsuchiura, Lake Kasumigaura | 2/8/08 | **217** | 19 | 68 | 105 | 94 | 66 | 62 | 59 | G |
| Ks08TS08 | Tsuchiura, Lake Kasumigaura | 2/8/08 | **218** | 35 | 59 | 62 | 49 | 54 | 96 | 25 | B |
| Ks08YA04 | Yasuzuka, Lake Kitaura | 3/8/08 | **117** | 45 | 66 | 75 | 52 | 63 | 62 | 59 | G |
| Ks08YA05 | Yasuzuka, Lake Kitaura | 3/8/08 | **117** | 45 | 66 | 75 | 52 | 63 | 62 | 59 | G |
| Ks08YA06 | Yasuzuka, Lake Kitaura | 3/8/08 | **117** | 45 | 66 | 75 | 52 | 63 | 62 | 59 | G |
| Ks08YA07 | Yasuzuka, Lake Kitaura | 3/8/08 | **117** | 45 | 66 | 75 | 52 | 63 | 62 | 59 | G |
| Ks08YA11 | Yasuzuka, Lake Kitaura | 3/8/08 | **219** | 45 | 67 | 127 | 66 | 63 | 97 | 59 | G |
| Ks08YA14 | Yasuzuka, Lake Kitaura | 3/8/08 | **220** | 47 | 86 | 85 | 94 | 105 | 68 | 88 | NA |
| Ks08YA15 | Yasuzuka, Lake Kitaura | 3/8/08 | **221** | 19 | 70 | 128 | 55 | 106 | 62 | 59 | G |
| Ks08YA16 | Yasuzuka, Lake Kitaura | 3/8/08 | **222** | 19 | 74 | 74 | 52 | 65 | 62 | 59 | G |
| Ks08YA19 | Yasuzuka, Lake Kitaura | 3/8/08 | **219** | 45 | 67 | 127 | 66 | 63 | 97 | 59 | G |
| Ks08YA20 | Yasuzuka, Lake Kitaura | 3/8/08 | **129** | 19 | 80 | 79 | 60 | 63 | 60 | 59 | G |
| Ks08YA22 | Yasuzuka, Lake Kitaura | 3/8/08 | **223** | 19 | 70 | 75 | 55 | 106 | 62 | 59 | G |
| Ks08YA25 | Yasuzuka, Lake Kitaura | 3/8/08 | **224** | 19 | 119 | 123 | 52 | 66 | 62 | 59 | G |
| Ks08YA26 | Yasuzuka, Lake Kitaura | 3/8/08 | **115** | 45 | 69 | 75 | 52 | 64 | 62 | 59 | G |
| As08Gb01 | Ogata-bashi, Hachiro Lagoon | 22/8/8 | **225** | 60 | 120 | 126 | 95 | 89 | 86 | 87 | NA |
| As08Gb02 | Ogata-bashi, Hachiro Lagoon | 22/8/8 | **226** | 64 | 2 | 2 | 2 | 28 | 2 | 89 | NA |
| As08Gb04 | Ogata-bashi, Hachiro Lagoon | 22/8/8 | **227** | 19 | 121 | 123 | 52 | 65 | 60 | 59 | G |
| As08Gb10 | Ogata-bashi, Hachiro Lagoon | 22/8/8 | **228** | 49 | 122 | 121 | 37 | 107 | 78 | 90 | NA |
| Aw08Gb01 | Ogata-bashi, Hachiro Lagoon | 22/8/8 | **229** | 66 | 123 | 129 | 13 | 108 | 98 | 47 | A |
| Aw08Fu08 | Funakoshi, Hachiro Lagoon | 22/8/8 | **161** | 28 | 91 | 97 | 73 | 80 | 74 | 68 | F |
| Aa08Fu02 | Funakoshi, Hachiro Lagoon | 22/8/8 | **232** | 67 | 81 | 52 | 61 | 90 | 48 | 61 | NA |
| Ai08Fu01 | Funakoshi, Hachiro Lagoon | 22/8/8 | **233** | 68 | 100 | 131 | 97 | 110 | 22 | 72 | NA |
| As08Fu02 | Funakoshi, Hachiro Lagoon | 22/8/8 | **233** | 68 | 100 | 131 | 97 | 110 | 22 | 72 | NA |
| As08Fu06 | Funakoshi, Hachiro Lagoon | 22/8/8 | **234** | 66 | 124 | 129 | 13 | 109 | 100 | 47 | A |
| Aw08Fu01 | Funakoshi, Hachiro Lagoon | 22/8/8 | **235** | 69 | 126 | 25 | 73 | 76 | 74 | 68 | F |
| Aw08Fu02 | Funakoshi, Hachiro Lagoon | 22/8/8 | **235** | 69 | 126 | 25 | 73 | 76 | 74 | 68 | F |
| An08Hj03 | Hojo, Hachiro Lagoon | 22/8/8 | **230** | 66 | 124 | 129 | 13 | 109 | 99 | 47 | A |
| As08Hj03 | Hojo, Hachiro Lagoon | 22/8/8 | **231** | 60 | 125 | 130 | 96 | 89 | 62 | 87 | NA |
| Ai08Si01 | Shin-imagata, Hachiro Lagoon | 22/8/8 | **236** | 20 | 127 | 132 | 75 | 33 | 24 | 6 | B |

a. Geographic location of NIES and TAC strains will be available at http://mcc.nies.go.jp/distribution-phylogeny_e.html. Other strains isolated by YT have been deposited in MCC-NIES (Tsukuba, Japan) and their information will be also available at the same web site.

b. Date of isolation (day/month/year). *,unknown.

c. Sequence type. ST1–102, 109–161 are according to [4-6].

d. Group assignment on the basis of the MLST phylogeny (Fig. 1). NA, strains assigned to neither group.
