# Supplementary material for: Local Expansion of a Panmictic Lineage of Water Bloom-Forming Cyanobacterium Microcystis aeruginosa
Source: PLoS One. 2011 Feb 24;6(2):e17085. doi: 10.1371/journal.pone.0017085 (PMC3044731; doi:10.1371/journal.pone.0017085)
Supplement: Table S2 — Genetic diversity and recombination. The results of maximum likelihood tests for tree congruence proposed by Feil et al. [9] supported the recombinogenic nature of the five lineages, where we observed a higher proportion of phylogenetic congruence between the phylogenetic tree of each locus and randomized ones. The estimated parameter values for relative impact (r/m) and rate (ρ/θ) of recombination and mutation also indicated the higher impact of recombination groups D and G1, where we observed higher values than for other lineages. On the other hand, both parameter estimates for groups C and F were less than 1, suggesting the lower frequency of recombination relative to point mutation. On the basis of the results obtained from analysis using the entire data set, we employed a constant value for the mean tract length of imported gene segments (δ = 225 bps) to infer the recombination parameters for each lineage. Because this value is much smaller than the length of all MLST loci (>400 bps), we probably failed to capture some of the entire-allele recombinational replacements. Thus, the inconsistency between the results of the LD analysis and recombination parameter inference could be due to the underestimation of the relative rate of recombination. Reference: Didelot X, Falush D (2007) Inference of bacterial microevolution using multilocus sequence data. Genetics 175: 1251–1266. Swofford DL (2002) PAUP* – Phylogenetic analysis using parsimony (*and other methods), version 4. Sunderland, MA: Sianuer Associates. Tajima F (1989) Statistical method for testing the neutral mutation hypothesis by DNA polymorphism. Genetics 123: 585–595. (DOC) [file pone.0017085.s004.doc]

**Table S2.** Genetic diversity and recombination.

| **Group** | ***n***^a^ | ***D***^b^ | ***r/m***^c^ | ***ρ/θ***^d^ | **Portion of significant congruence** ^e^ |
| --- | --- | --- | --- | --- | --- |
| A | 50 | 0.553 | NA^f^ | NA^f^ | 42/42 |
| B | 54 | 0.434 | 1.846 | 0.713 | 29/42 |
| C | 6 | 0.084 | 0.206 | 0.047 | 5/25 |
| D | 12 | 0.725 | 2.471 | 0.976 | 1/16 |
| E | 13 | 0.028 | ND^g^ | ND^g^ | 1/25 |
| F | 6 | -0.046 | 0.216 | 0.037 | 4/36 |
| G | 44 | 0.149 | 1.671 | 0.803 | 12/42 |
| G1 | 40 | 1.088 | 4.357 | 2.878 | 11/42 |
| G2 | 24 | 0.102 | ND^g^ | ND^g^ | 3/36 |
| Other | 52 | 0.319 | NA^f^ | NA^f^ | NT |
| Total | 237 | 0.353 | 2.895 | 1.153 | 42/42 |

a. Number of ST.

b. Tajima's *D* (Tajima, 1989) using unique STs, calculated by DnaSP. All values are not significantly different from zero (*P* > 0.10).

c. Relative contribution of recombination to point mutation to the observed genetic diversity per site inferred using ClonalFrame (Didelot and Falush, 2007). The values were based on the concatenation of half or third of 100000-300000 iterations of 5-15 independent runs of ClonalFrame. For the analyses of each group, the mean tract length of imported gene segments was not jointly inferred and the constant value (*δ* = 225 bps), which was inferred from the analysis of the whole dataset, was used. The convergence of MCMC chains in different runs was assessed by Gelman-Rubin test implemented in the ClonalFrame and Tracer version 1.5 (Rambaut A, Drummond AJ, available from http://beast.bio.ed.ac.uk/Tracer), under the criteria of Gelman-Rubin statistics < 1.2 and ESS > 200, respectively, as suggested by the authors. NT, not tested.

d. Relative recombination rate scaled by mutation rate inferred by ClonalFrame. The values were based on the same method as in *r*/*m*. NT, not tested.

e. Maximum likelihood (ML) analysis of tree topology congruence proposed by Feil *et al*. [9] using PAUP* version 4.0b10 (Swofford, 2002). The log-likelihoods of 200 random trees were calculated based on the sequence data of each locus, and compared to those of the ML tree topologies of the other six loci. Log likelihood scores higher than those of 99th percentile of 200 random tree topologies are "significantly congruent" (*P* < 0.01), suggesting lack of recombination. Thus, the lower value of the portion of significant congruence indicate the higher rate of recombination. NT. Not tested.

f. NA, not analysed because these groups are polyphyletic in ClonalFrame analyses.

g. ND, not determined due to poor convergence between independent MCMC chains of ClonalFrame analysis.

**Table S2 legends**

The results of maximum likelihood tests for tree congruence proposed by Feil *et al.* [9] supported the recombinogenic nature of the five lineages, where we observed a higher proportion of phylogenetic congruence between the phylogenetic tree of each locus and randomized ones. The estimated parameter values for relative impact (*r/m*) and rate (*ρ/θ*) of recombination and mutation also indicated the higher impact of recombination groups D and G1, where we observed higher values than for other lineages. On the other hand, both parameter estimates for groups C and F were less than 1, suggesting the lower frequency of recombination relative to point mutation. On the basis of the results obtained from analysis using the entire data set, we employed a constant value for the mean tract length of imported gene segments (*δ* = 225 bps) to infer the recombination parameters for each lineage. Because this value is much smaller than the length of all MLST loci (> 400 bps), we probably failed to capture some of the entire-allele recombinational replacements. Thus, the inconsistency between the results of the LD analysis and recombination parameter inference could be due to the underestimation of the relative rate of recombination.

**Reference**

Didelot X, Falush D (2007) Inference of bacterial microevolution using multilocus sequence data. Genetics 175: 1251–1266.

Swofford DL (2002) *PAUP* – Phylogenetic analysis using parsimony (*and other methods)*, version 4. Sunderland, MA: Sianuer Associates.

Tajima F (1989) Statistical method for testing the neutral mutation hypothesis by DNA polymorphism. Genetics 123: 585–595.
