# Supplementary material for: Local Expansion of a Panmictic Lineage of Water Bloom-Forming Cyanobacterium Microcystis aeruginosa
Source: PLoS One. 2011 Feb 24;6(2):e17085. doi: 10.1371/journal.pone.0017085 (PMC3044731; doi:10.1371/journal.pone.0017085)
Supplement: Table S3 — Result of Ecotype Simulation. The values in parenthesis indicate 95% confidence intervals for each parameter estimated. Note that ecotype demarcation analysis implemented in Ecotype Simulation software conservatively identified 85 putative ecotypes, which was lesser than that estimated by simulation analysis. However, the result indicated many more ecotypes than expected. Nevertheless, groups G1 and G2 without G1 were predicted as ecotypes (see also Fig. 1). (DOC) [file pone.0017085.s005.doc]

**Table S3.** Result of Ecotype Simulation.

| **Clade** | **Net rate of ecotype formation**  **(Ω)** | **Rate of periodic selection**  **(σ)** | **Rate of drift**  **(d)** | **Ecotypes**  **(n)** |
| --- | --- | --- | --- | --- |
| Group A to G and X | 0.10976  (0.10976, 0.10976) | 0.558  (0.110, >100) | 0  (0, ∞ ) | 190  (4, 190) |

**Table S3 legends**

The values in parenthesis indicate 95% confidence intervals for each parameter estimated. Note that ecotype demarcation analysis implemented in Ecotype Simulation software conservatively identified 85 putative ecotypes, which was lesser than that estimated by simulation analysis. However, the result indicated many more ecotypes than expected. Nevertheless, groups G1 and G2 without G1 were predicted as ecotypes (see also Fig. 1).
